# Structure–Property Correlations in Disubstituted 1,2,3-Triazoles: DFT Insights and Photophysical Analysis

**DOI:** 10.1021/acsomega.5c07623

**Published:** 2025-11-24

**Authors:** Dharatiben Lakhani, Sheeba Sadiq, Harini Subbaiahgari, Violet Swanson, Jacob Munyon, Sher B. Poudel, Karelle S. Aiken, Shainaz M. Landge, Debosreeta Bose, Debanjana Ghosh

**Affiliations:** † Department of Chemistry, 7604 Southern Illinois University Edwardsville, Science Building West, Box -1652, Edwardsville, Illinois 62026-1652, United States; ‡ Department of Biochemistry, Chemistry, and Physics 3728 Georgia Southern University (Statesboro Campus), 521 College of Education Drive, Statesboro, Georgia 30460-8064, United States; § Department of Chemistry, Amity Institute of Applied Sciences (AIAS), 530170 Amity University - Kolkata Campus, Rajarhat, Newtown, Kolkata, West Bengal 700135, India

## Abstract

The inherent structural rigidity and modifiable electronic features of disubstituted 1,2,3-triazoles are driving their recognition as adaptable frameworks in photophysical and supramolecular chemistry. The combination of structural integrity and tunable electronic characteristics makes 1,2,3-triazoles highly functional for a wide range of applications, from fluorescence-based sensors and drug design to organic electronics. Owing to the versatile applications of triazoles in multiple domains, this study integrates experimental and computational analysis of a series of 1,4- and 1,5-disubstituted 1,2,3-triazole derivatives. To elucidate the electronic architecture, photophysical characteristics, and structure–property correlations, 1,2,3-triazoles with and without a hydroxyaromatic framework have been investigated. Emphasis has been given to the molecules’ solvatochromic behavior in medium-polarity solvents. The photophysical behavior of the studied 1,2,3-triazole molecules is also modulated by pH, reflecting changes via a protonation/deprotonation pathway specific to the individual molecules. Systematic spectroscopic investigations revealed differential absorption and emission responses across acidic to basic environments, highlighting the sensitivity of triazole chromophores to proton-coupled electronic interactions. Density Functional Theory (DFT) and Time-Dependent DFT (TD-DFT) calculations at the B3LYP/6-311G­(d,p) level were employed to investigate the frontier molecular orbitals (FMOs), electron density distributions, and polarity of the studied molecules, with outcomes corroborated by UV–vis and fluorescence spectroscopic measurements. Additionally, Natural Bond Orbital (NBO) analysis is performed for 2-(4-phenyl-1-H-1,2,3-triazol-1-yl) phenol (PTP) and 5-anilino-4-phenyl-1H-1,2,3-triazole (APT), representing hydroxyaromatic and nonhydroxyaromatic triazoles, respectively. This analysis primarily focuses on establishing the photophysical characteristics of the molecules as being of a charge-transfer nature and their inherent electron density distribution. A comparative analysis of experimental and computational data revealed a consistent and complementary trend, reinforcing the reliability of the interpretations drawn from both approaches.

## Graphical abstract

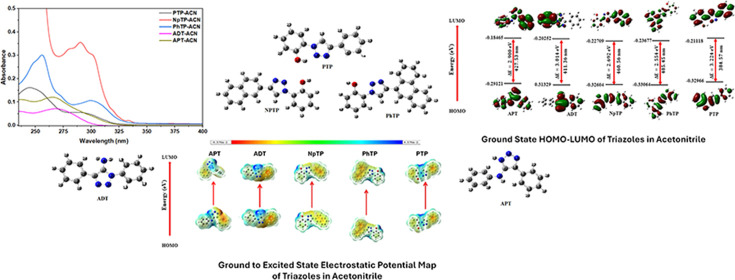

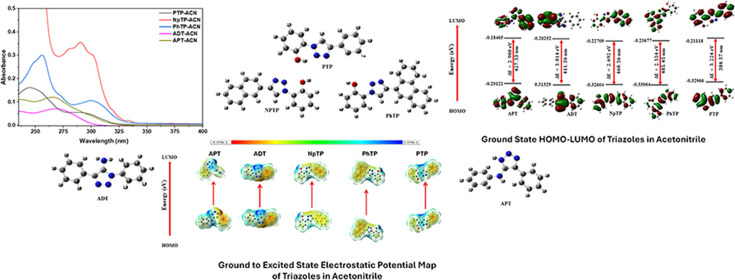

## 1 Introduction

The five-membered heterocycles with aromatic character present a versatile group of compounds possessing rich chemical functionality. In particular, 1,2,3-triazoles have emerged as valuable components in the design of organic dyes, fluorescent metal ion sensors, OLED materials, corrosion inhibitors, and catalysts.^1,2^ Among their structural isomers, the 1,4- and 1,5-disubstituted 1,2,3-triazoles have gained widespread attention due to their ease of synthesis, regioselectivity, and broad applicability across disciplines. The classic *Click chemistry* approach through 1,3-dipolar cycloaddition has enabled the streamlined synthesis of a broad spectrum of functional 1,2,3-triazole molecules for various applications, such as in medicinal chemistry, materials science, bioconjugation, and organic electronics.^3^ Further potential to control the electronic and steric makeup is offered by methods for the selective preparation of either 1,4- or 1,5-substituted triazoles during azide–alkyne cycloaddition.^4^

Beyond their structural utility, 1,2,3-triazole derivatives have demonstrated promising photophysical properties,^5^ making them attractive as fluorophores in chemical and biological sensing. Kautny and coworkers^4^ employed click chemistry to synthesize a series of 1,2,3-triazole-linked donor–acceptor chromophores, systematically investigating how variations in acceptor strength via sulfur oxidation, double bond geometry, and triazole substitution patterns influence the nature of charge-transfer states in these conjugated systems. A new series of 1,2,3-triazole hybrids was synthesized where 2- or 4-hydroxyphenyl benzothiazole (HBT), naphthalen-1-ol, or 8-hydroxyquinoline (8-HQ) was incorporated.^6^ Among them, quinoline- and 2-HBT-linked derivatives with short alkyl linkers showed the highest DNA binding affinity driven by hydrogen bonding and hydrophobic forces.

Numerous studies on the triazoles have documented their ability to detect a variety of analytes through fluorescence modulation, capitalizing on their electronic tunability and ability to participate in noncovalent interactions.^7^ These heterocycles also offer useful sites for hydrogen bonding and π-interactions, further enhancing their behavior in supramolecular environments. Recently, triazole-containing hybrids, including those conjugated to extended π-systems like indolizines or hydroxyaromatics, have shown potential for environment-sensitive fluorescence, excited-state intramolecular proton transfer (ESIPT), and tunable emission behavior.^8^ Previous experiments from our group on 1,4-disubstituted 1,2,3-triazoles demonstrated that the ortho-hydroxy phenolic groups on the triazole core act as effective anion sensors, showing a *turn-on* fluorescence or a change in signal output and 1:1 binding stoichiometry with fluoride (F^–^).^9–13^ During analyte binding, the probe adopted a nearly planar conformation, suggesting that the fluoride engages in hydrogen bonding with the phenolic −OH group, which is positioned close to the triazole Csp^2^ −H proton. Analytical findings indicate that this hydrogen bonding interaction plays a pivotal role in initiating the deprotonation process. Incorporation of electron-donating amino (−NH_2_) groups improved selectivity toward F^–^ over H_2_PO_4_^–^ and AcO^–^.^9^ A different observation was made when bis-triazole (BPT) system^14^ and phenanthrene-triazole (PhTP) derivative^10^ were subjected to metal ion recognition. Cu^2+^ induced a visible color change under ambient light and led to fluorescence quenching, enabling dual-mode detection. These findings support the design of molecular logic gates based on triazole-based fluorophores for smart sensing applications.^15^

In contrast to the widely studied 1,4-disubstituted triazoles, the 1,5-disubstituted isomers exhibit significantly greater steric crowding due to the shorter distance between substituents on the triazole ring (approximately 2.4 Å in 1,5- vs 5.0 Å in 1,4-disubstituted structures).^16^ Structurally, the 1,4-disubstituted 1,2,3-triazole isomer closely simulates the geometry of a trans-amide bond, whereas the 1,5-disubstituted isomer resembles a cis-amide bond conformation. The substitution pattern at vicinal nitrogen and carbon positions in 1,5-disubstituted triazoles imposes conformational constraints which enhance its potential for designing biomolecular mimetics, such as macrocyclic architectures and analogs of natural products, where spatial constraints and conformational rigidity are critical for function.^17^ The 1,5-disubstituted 1,2,3-triazole ring is a nitrogen-rich heterocycle with a high dipole moment and a rigid, planar structure that facilitates well-defined noncovalent interactions, including hydrogen bonding and dipole–dipole interactions. H-bonding is one of the essential noncovalent forces that play an important role in the structure–function property of proteins. This characteristic of the triazole derivative plays a key role in molecular recognition processes and contributes to the stabilization of protein secondary structures.^18,19^

Herein, we report on a fundamental study aimed at revisiting and deepening our understanding of the photophysical properties of selected 1,2,3-triazole derivatives in various solvent environments. In this work, 1,4-substituted triazoles with a hydroxyaromatic framework, such as 2-(4-phenyl-1-H-1,2,3-triazol-1-yl) phenol (PTP), 2-(4-(naphthalen-2-yl)-1H-1,2,3-triazol-1-yl) phenol (NpTP) and (2-[4-(phenanthren-9-yl)-1H-1,2,3-triazol-1-yl]­phenol (PhTP), and 1,5-substituted triazoles with nonhydroxyaromatic framework, such as 5-amino-1,4-diphenyl-1,2,3-triazole (ADT) and 5-anilino-4-phenyl-1H-1,2,3-triazole (APT) are studied (Scheme 1). Our interest in these scaffolds lies in their potential for chemosensing applications, specifically for their ability to detect environmentally and biologically relevant ions such as fluoride and copper­(II).^9–15,20^ For this reason, a deeper understanding of how the molecules themselves behave in different solvents and the capability to predict their behaviors will benefit the long-term goals of our work. Owing to their distinct structural frameworks, these molecules offer a versatile platform for investigating how subtle structural modifications impact emission behavior and solvatochromic responses, guided by their inherent electronic tunability and conformational rigidity. By systematically analyzing their UV–vis absorption and fluorescence characteristics across solvents of varying polarity, we aim to elucidate how environmental factors and molecular design govern excited-state processes, such as charge transfer, hydrogen bonding, and dipolar interactions. To complement the experimental findings, we employ computational chemistry techniques, including density functional theory (DFT) and time-dependent DFT (TD-DFT), to probe the electronic structures, frontier molecular orbitals (FMOs), and excited-state transitions of these triazole systems. These calculations allow us to draw structure–property correlations and predict how specific modifications, such as substitution pattern, electron-donating or withdrawing groups, and solvent polarity, affect the photophysical behavior. The combined experimental–theoretical approach enables a more nuanced understanding of structure–function relationships, providing a rational basis for molecular design.

**Scheme 1:**
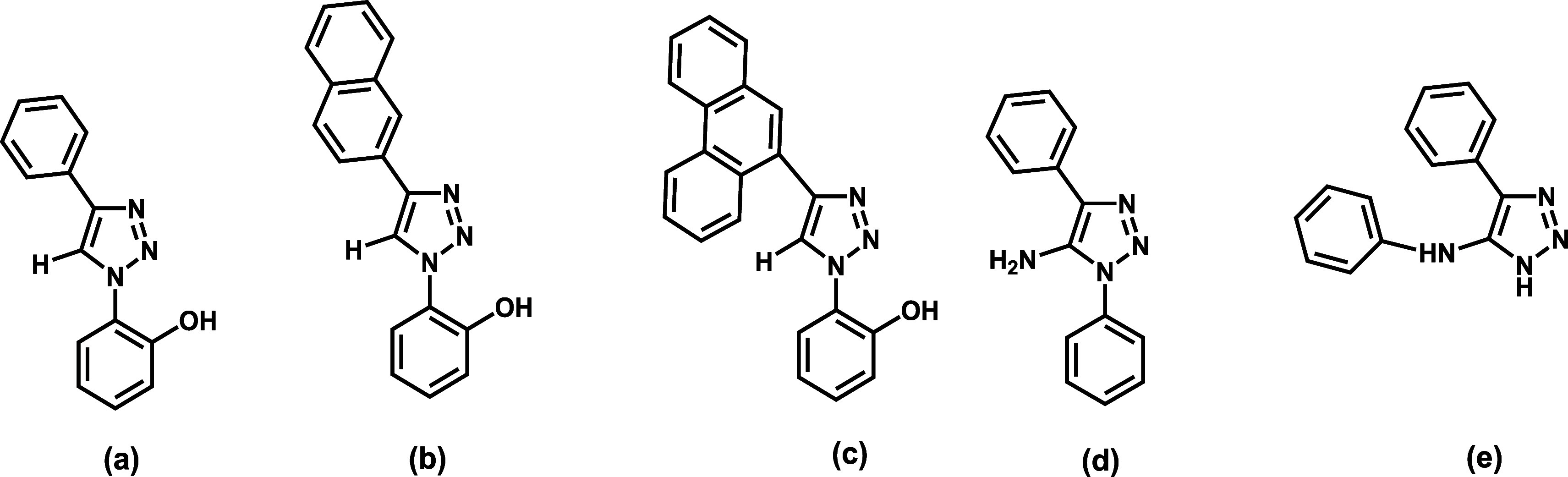
Structures of (a) PTP, (b) NpTP, (c) PhTP, (d) ADT, and (e) APT

The insights gained from this study will have broader implications for the development of functional host–guest systems, where noncovalent interactions, fluorescence switching, and responsive emission are the key. Understanding how the triazole framework behaves in different environments will inform future efforts to design supramolecular sensors, molecular logic gates, or stimuli-responsive materials. Ultimately, this work will contribute to the foundational knowledge needed to tailor 1,2,3-triazole-based architectures for diverse applications in supramolecular chemistry, chemical sensing, and smart material design.

## 2 Results and Discussion

To explore the photophysical behavior of 1,2,3-triazole derivatives, we investigated structurally diverse 1,4- and 1,5-disubstituted analogs, leveraging their rigid backbones and electronically tunable frameworks. Spectroscopic measurements (UV–vis and fluorescence) were complemented by DFT and TD-DFT calculations to examine how substitution patterns and the presence of hydroxyaromatic groups influence electron density distribution, frontier orbital characteristics, and emission behavior.

### 2.1 Photophysical Studies

#### 2.1.1 UV–Vis Absorption

Steady-state UV–vis absorption spectra of all compounds, PTP, NpTP, PhTP, ADT, and APT, were recorded in acetonitrile (ACN) for two purposes: first, to check the consistency of the earlier reported data of the hydroxyaromatic triazoles, and second, to investigate the influence of the structural modifications (nonhydroxyaromatic) on the molecular properties of the materials.^10,12,13^ The 1,4-disubstituted triazoles with the hydroxyaromatic framework, PTP, NpTP, and PhTP, exhibited similar absorption profiles across (Figure 1) with two prominent transitions. A low-energy absorption peak attributed to the π-π* transition appears around 285 nm for these molecules in ACN. NpTP showed a structured absorption compared to the other analogs of the polyaromatic ring system. The nonhydroxyaromatic triazole ADT (Figure 1) showed a prominent transition centered around the maximum absorption peak at 270 nm. In the UV–vis spectrum of APT (Figure 1), another of the nonhydroxyaromatic triazoles and the isomer of ADT,^21–24^ the absorption peak was observed around 260–270 nm, consistent with ADT. However, a hump around 300 nm in the absorption spectrum was further noticed, favoring a n→π* transition due to the presence of the anilino group at the 5-position of the 1,2,3-triazole core. The high-energy band present in all the molecules is observed as a characteristic shoulder around 250 nm.

**Figure 1:**
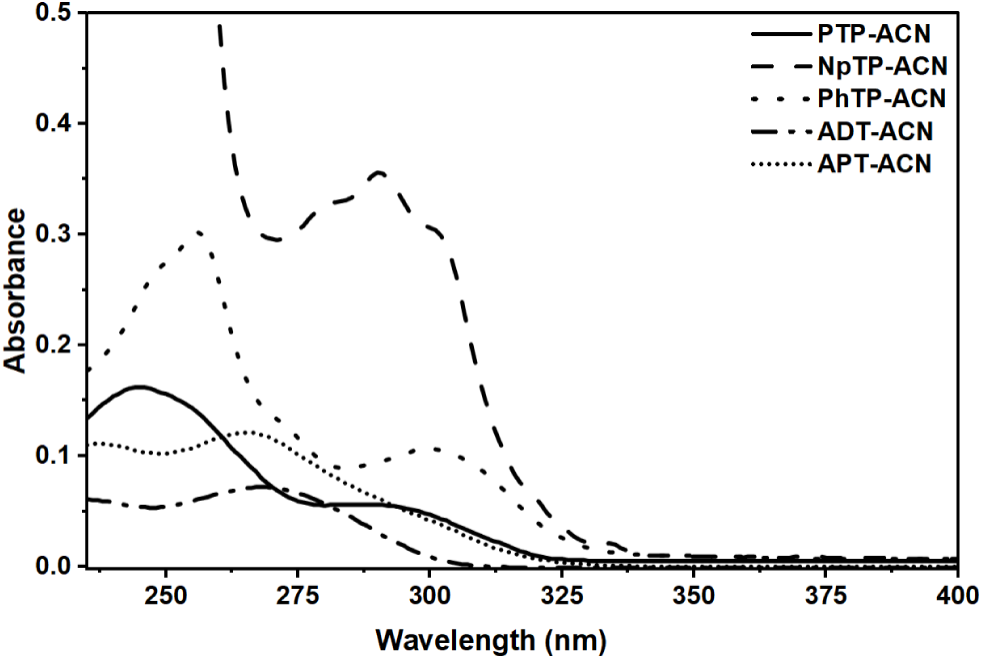
Absorption spectra of 1,2,3-triazoles (indicated by the legends) in ACN solid line for PTP, dashed line for NpTP, dotted line for PhTP, dashed-dotted line for ADT, and short-dotted line for APT).

#### 2.1.2 Fluorescence

Distinct fluorescence profiles were observed for the 1,2,3-triazole derivatives under investigation (Figure 2). The parent compound, PTP, is inherently nonemissive (Figure 2a). However, structural modification to NpTP and PhTP, featuring a naphthalene and phenanthrene substituent, respectively, at the 4-position of the triazole core, yielded a markedly different emission behavior. Upon exciting NpTP at 300 nm, a structured emission band (Figure 2b) spanning between 345 and 380 nm was attributed to the naphthalene moiety. PhTP, on the other hand, exhibited a pronounced fluorescence signature characteristic of the phenanthrene chromophore, with steady-state emission spanning 335–500 nm (Figure 2c). The emission was notably intense, accompanied by a high fluorescence yield (*vide* Table S6 for quantum yield), highlighting the role of extended conjugation in modulating excited-state properties. Similar to PTP, ADT, the nonhydroxyaromatic 1,5-disubstituted triazole derivative displayed inappreciable fluorescence (Figure 2d) in ACN, indicating limited delocalization or inefficient excited-state relaxation pathways in these structures. Interestingly, the 5-anilino substituted triazole, APT, demonstrated a significantly different photophysical response (Figure 2e). Excitation of APT at 270 nm in ACN produced a broad, featureless emission centered around 390 nm. This unstructured fluorescence profile, along with the considerable Stokes shift (Table S6), suggested efficient intramolecular charge redistribution and electronic relaxation within a rigid, conjugated aromatic framework.

**Figure 2:**
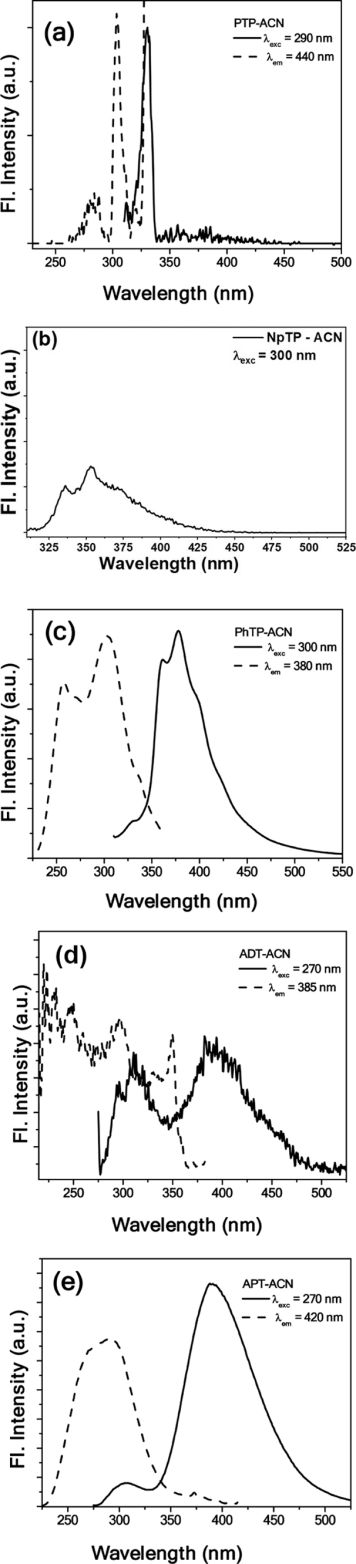
Emission (solid lines) and excitation (dashed lines) spectra of 1,2,3-triazoles, (a) PTP (λ_ex_ = 290 nm), (b) NpTP (λ_ex_ = 300 nm), (c) PhTP (λ_ex_ = 300 nm), (d) ADT (λ_ex_ = 270 nm), and (e) APT (λ_ex_ = 270 nm) in ACN. Concentrations of the molecules for collecting fluorescence are 2 × 10^–5^ M for PTP, 1 × 10^–5^ M for NpTP and PhTP, while it is 2 × 10^–6^ M for ADT and APT.

The structural origin of APT and ADT can be traced back to a model synthetic route developed by Lieber et al. in the 1950s, wherein 1,4-disubstituted-5-amino-1,2,3-triazoles undergo irreversible isomerization to yield 4-phenyl-5-(substituted)­anilino-1,2,3-triazoles upon refluxing in pyridine-type bases.^24^ This transformation proceeds via the well-established Dimroth rearrangement mechanism,^25^ confirming that ADT and APT are constitutional isomers interconvertible under specific conditions. Despite being isomeric, ADT and APT exhibited markedly different electronic configurations, which is manifested as divergent excited state photophysical behaviors. These results emphasize the sensitivity of fluorescence to subtle electronic and structural variations within the triazole scaffold, offering insights into the design of functional, emissive materials based on this heterocyclic core.

### 2.2 Solvatochromism of Triazoles

The solvatochromic behavior of the 1,2,3-triazoles was investigated to provide a better insight into the nature of the excited states of the compounds and to examine the impact of structural modifications on the photophysical properties of individual molecules. Absorption and emission spectra were recorded for PTP and PhTP of the hydroxyaromatic compounds (1,4-disubstituted) and two nonhydroxyaromatic analogs (1,5-disubstituted), ADT and APT, across a range of solvents with varying polarity and proticity, e.g., water, methanol, ethanol, ACN, hexane, and heptane. It is expected that if the interaction occurs in the ground state, then some change in the absorption spectrum will be noticed.

#### 2.2.1 Absorption Studies

The UV–vis absorption profile of PTP (Figure 3a) exhibited a notable blue shift in water (∼280 nm), accompanied by reduced optical density, compared to its absorption in ACN (∼292 nm). As the solvent polarity decreased from ACN to hexane, the absorption maxima red-shifted slightly, appearing at ∼303 nm, with a corresponding increase in absorbance. The red shift observed is due to the π–π* excitation state being very stable in aprotic solvents, leading to a decrease in the energy gap between the two states. In comparatively less polar, more polarizable solvents, such as ethanol and hexane, excited-state stabilization through dispersion and polarizability caused absorption at longer wavelengths.^26^ PhTP followed a similar trend across the same solvent series, indicating a consistent solvatochromic response linked to its hydroxyaromatic framework (Figure 3b).

**Figure 3:**
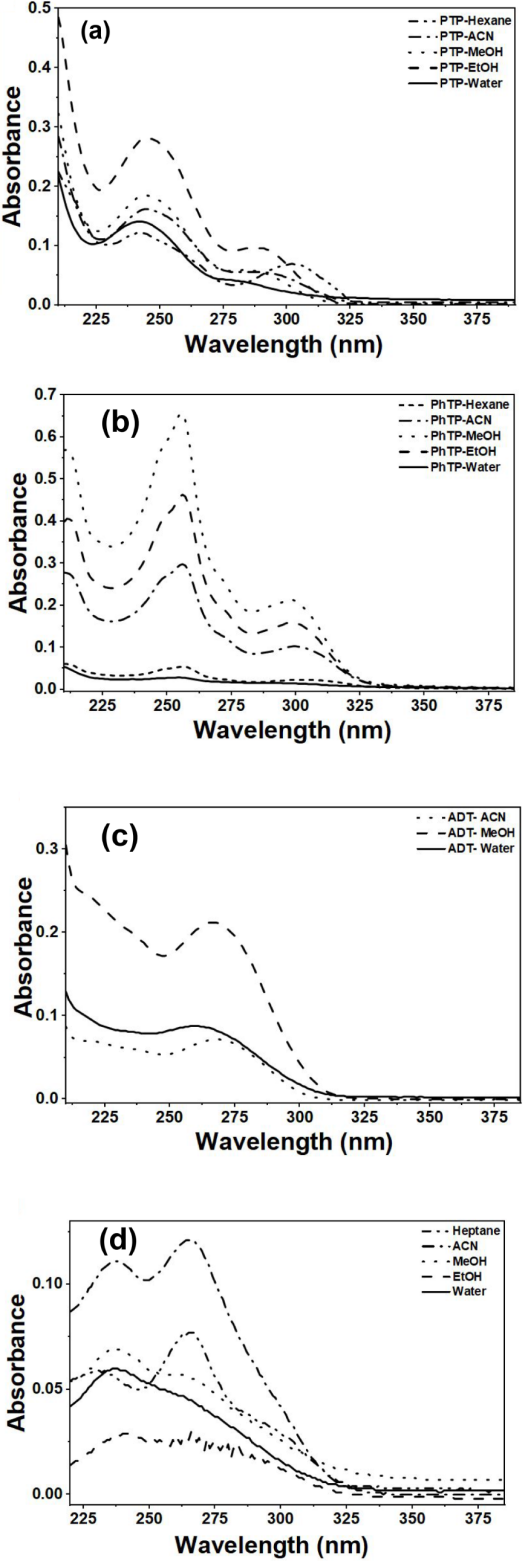
Absorption spectra of 1,2,3-triazoles, (a) PTP, (b) PhTP, (c) ADT, and (d) APT in different solvents.

In contrast, the nonhydroxyaromatic compound ADT displayed a more subtle absorption behavior shaped by both solvent polarity and proticity (Figure 3c). A hypsochromic shift was observed from 270 nm in ACN to 265 nm in methanol and 260 nm in water, suggesting stronger ground-state stabilization in polar protic solvents. APT, another nonhydroxyaromatic derivative, showed minimal change in absorption maxima across solvents, a behavior characteristic of certain triazole-containing systems (Figure 3d). However, its spectral profile portrayed a solvent-dependent variations in vibronic transitions. In aprotic solvents such as ACN and heptane, the absorption peaks around 265 nm appeared sharper and more intense than in polar protic solvents (e.g., ethanol, methanol, and water), suggesting solvent-specific interactions influencing spectral fine structure. Overall, across all the triazole compounds, solvents with medium polarity indices (ranging from 5.1 to 5.8) consistently exhibited either a significant spectral shift or enhanced absorbance as compared to the rest. Table S6 reflects the absorption maxima of each 1,2,3-triazole in different solvents.

#### 2.2.2 Fluorescence Studies

Photoexcitation in these solvents revealed distinct emission behaviors. PTP exhibited negligible fluorescence in low-polarity aprotic solvents but showed weak emission in polar protic media like methanol, ethanol, and water (Figure 4a). In low-polarity environments, the lack of strong solute–solvent interactions failed to adequately stabilize the excited state, resulting in rapid dissipation of the excitation energy through nonemissive pathways. Moreover, in such solvents, the π–π* or n−π* transitions typical of hydroxyaromatic compounds may undergo efficient internal conversion due to unrestricted intramolecular motions or torsional flexibility, leading to fluorescence quenching.^27–29^ However, when PTP is dissolved in polar protic solvents such as methanol, ethanol, and water, a weak but noticeable emission is observed. This suggests that hydrogen bonding and dipole–dipole interactions between the solvent molecules and the hydroxyl functional group of PTP may help stabilize the excited state and suppress nonradiative decay. Polar protic solvents can form hydrogen bonds with the hydroxyl moieties, potentially restricting vibrational and rotational degrees of freedom in the excited state. The enhancement in PTP’s quantum yield was also noticed when the solvents changed from ACN to more polar protic ones (Table S6a), corroborating the fact. Another of the same skeletal structure as PTP, PhTP’s emission spectra remained relatively unchanged in wavelength across different solvents (Figure 4b), reproducing the observation from the previously reported work.^20^ However, a marked drop in fluorescence was observed in water, indicating solvent-induced quenching (Table S6b).

**Figure 4:**
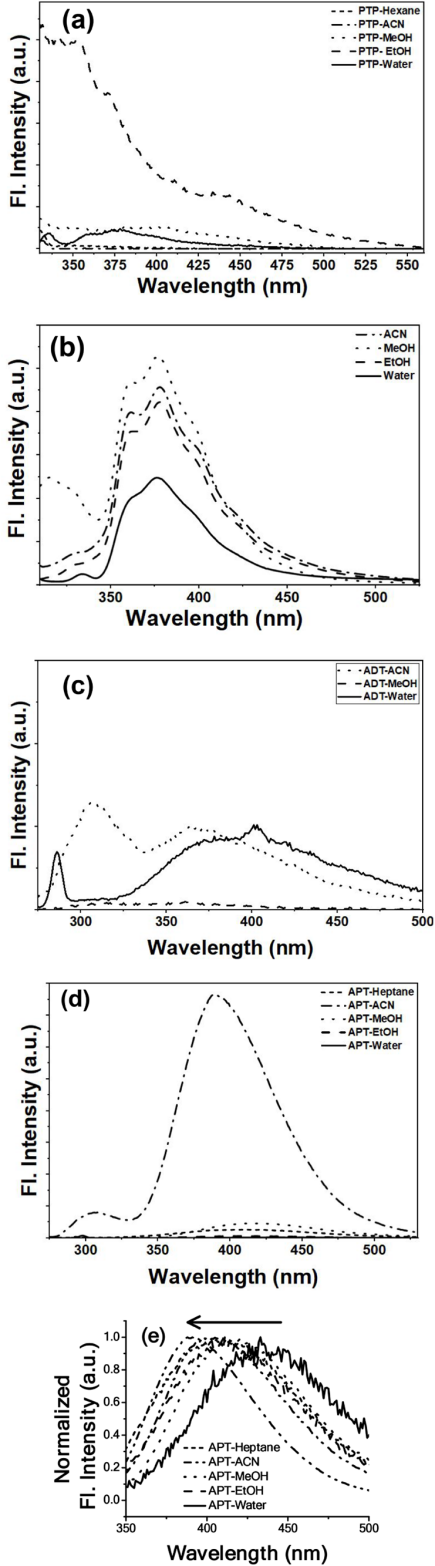
Emission spectra of 1,2,3-triazoles, (a) PTP (λ_ex_ = 290 nm), (b) PhTP (λ_ex_ = 300 nm), (c) ADT (λ_ex_ = 270 nm), and (d,e) APT (λ_ex_ = 270 nm) in different solvents are provided within the figure legends.

Of the nonhydroxyaromatic framework, ADT remains largely nonfluorescent in most solvents (Figure 4c), suggesting that nonradiative decay pathways dominate the excited-state relaxation. The absence of significant emission may be attributed to efficient internal conversion or intersystem crossing processes that compete with fluorescence. An evaluation of the quantum yield of ADT in the solvents revealed low values reflecting the observation (Table S6c). A modest enhancement in fluorescence intensity was observed in water, indicating that the solvent environment plays a role in modulating the excited-state dynamics. The slight emission increase in water may arise from solute–solvent interactions like hydrogen bonding or solvation effects involving the −NH_2_ group, which restricted nonradiative relaxation by limiting molecular motion or stabilizing the excited state. Additionally, the higher polarity and proticity of water may induce a modest redistribution of electron density in the excited state, potentially altering the Franck–Condon factors or oscillator strength to slightly favor radiative decay (*vide* section 2.4). These solvent-specific effects highlight how microenvironmental factors can influence emissive behavior even in inherently weakly fluorescent systems like ADT. Interestingly, an independent study with ADT (Figure S1) demonstrated that its fluorescence was significantly enhanced under acidic conditions, highlighting its pH-responsive nature (Section 2.3).

APT exhibited the most striking solvent-dependent fluorescence behavior (Figure 4d). A summary of the quantum yield of APT is provided in Table S6d. Upon excitation at 270 nm, APT showed its highest quantum yield in ACN (λ_em_ = 390 nm, Φ = 0.61), which is in stark contrast with water (λ_em_ = 435 nm, Φ = 0.009). Other polar protic solvents such as methanol (λ_em_ = 420 nm) and ethanol (λ_em_ = 411 nm) yielded substantially lower quantum yields, indicative of pronounced fluorescence quenching. While heptane produced a visibly intense emission, its moderate quantum yield (λ_em_ = 408 nm, Φ = 0.04) suggested that the luminescence could arise from suppressed nonradiative decay rather than from inherently high emission efficiency. The normalized emission spectra (Figure 4e) further highlighted solvatochromic shifts. While no uniform trend in emission maxima was apparent across all solvents, a general blue shift (hypsochromic) was noted from water to less polar solvents like ACN. Among the alcohol solvents, ethanol showed the most blue-shifted emission, followed by methanol. A direct comparison between heptane and ACN revealed a more pronounced blue shift in the latter. These solvent-induced spectral changes are consistent with differential stabilization of electronic states in which polar solvents preferentially stabilize the polar ground state via dipole–dipole interactions and hydrogen bonding, increasing the energy gap between ground and excited states and resulting in a blue shift in emission.^30^

#### 2.2.3 Viscosity Studies

Further to the solvatochromic interactions, PhTP and APT’s photophysics were independently observed in glycerol–water medium. An earlier study of PhTP in such a viscous medium^20^ provided insights into the molecule’s orientation in a rigid environment. With an increasing glycerol content in proportion to water, PhTP’s emission was seen to increase gradually with a slight red shift in the emission. A better resolution of the vibrational fine structures was also observed, correlating the molecule’s photophysics in a viscous medium. When APT’s absorption and fluorescence behavior were studied, the UV–vis absorption spectra (Figure S10a) of the molecule revealed an initial increase in the absorbance with increasing glycerol content (0–40% glycerol). A slight red shift from 265 to 270 nm and a gradual decrease in the absorption were noticed with higher glycerol (50–70%). Emission scans at λ_exc_ ∼ 270 nm (Figure S10b) showed a blue shift (440 to 425 nm) with increasing glycerol content. This trend matches APT's behavior in low-polarity solvents. The emission intensity, however, remained inconsistent across scans. Excitation scans, monitoring emission at 415 nm (Figure S10d), indicated the emergence of a new peak around 330 nm as glycerol in the medium increased. When APT was excited at this wavelength (λ_exc_ 330 nm) across varying glycerol–water compositions, a broad emission band was obtained, retaining the maximum emission, accompanied by an increase in fluorescence intensity (Figure S10c). A blue shift in the peak maximum (440 to 415 nm) was noted. This observation correlates with APT’s response in solvents. The overall observation accounts for a differential partitioning of APT in the glycerol–water mixture. The progressive increase in glycerol content creates a more rigid microenvironment, which suppresses intramolecular motion and slows molecular rotation, thereby enhancing the stability of the emissive state.

### 2.3 pH Response

The photophysical behavior of the 1,2,3-triazoles has been examined under a wide range of pH for PTP (Figure 5), PhTP (Figure S6), and APT (Figure 6) using UV–vis absorption and fluorescence spectroscopy. The lowest energy absorption band of PTP appeared around 280 nm at pH ∼ 1 (Figure 5). The characteristic absorption spectrum is significantly modulated upon increasing the pH. At first, within the pH range of 1.02–4.54, a small decrease in the absorption at 280 nm was noticed along with the development of a broad low absorbance band around 360 nm. The spectral characteristics of this broad band resemble the absorption profile of PTP observed in our previous study involving Cu^2+^, where the addition of Cu^2+^ led to a decrease in the local pH (pH 4.3) of the medium.^15^ This change in the spectra is attributed to the protonation of the triazole nitrogen in the molecule. Second, as the pH gradually increased from 6.05, a new peak developed around 302 nm with a concomitant decrease in the absorption at 280 nm. This observation is similar to our earlier study when PTP sensed F^–^ in ACN medium.^13^ A gradual addition of fluoride increased the local pH of the medium, facilitating the deprotonation of the phenolic −OH in the molecule. Emission spectra of PTP (Figure 5b) showed a maximum fluorescence at 425 nm at pH 1.02 on photoexcitation at 290 nm. Thereafter, an initial quenching of fluorescence and a blue shift was observed upon increasing the pH from 1.02 to 4.54. From pH 6.05 onward, a new peak gradually emerged around 475 nm. The broad band around 475 nm gained prominence in a moderately basic pH of 9.67 to 11.8. This observation in the emission profile is also similar to the previous study^13,15^ that hinted at the formation of the phenolate ion due to deprotonation of the hydroxyaromatic triazole molecule. The emission spectra for the other hydroxyaromatic analog, PhTP (Figure S6b), revealed an enhancement in fluorescence from pH 1.02 to 3.3 peaking at ∼390 nm (λ_exc_ = 300 nm). A gradual decrease in fluorescence was then observed at relatively higher pH, accompanied by a red shift (390 to 402 nm) in the spectra, an observation that attributes the deprotonation of the phenolic −OH present in the 1,4-disubstituted triazole molecules.

**Figure 5:**
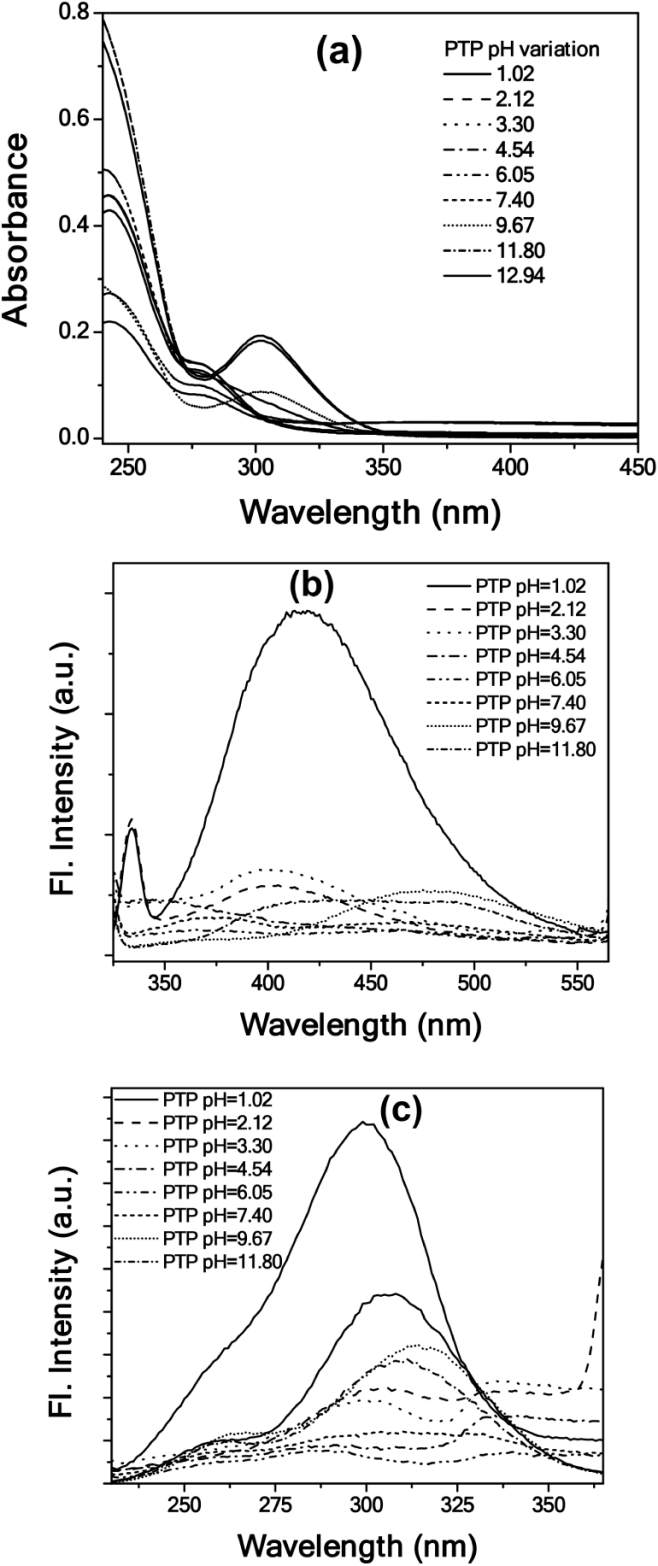
(a) UV–vis absorption, (b) emission (λ_exc_ = 290 nm), and (c) excitation (λ_em_ = 450 nm) spectra of PTP (2 × 10^–5^ M) at different pH levels.

**Figure 6:**
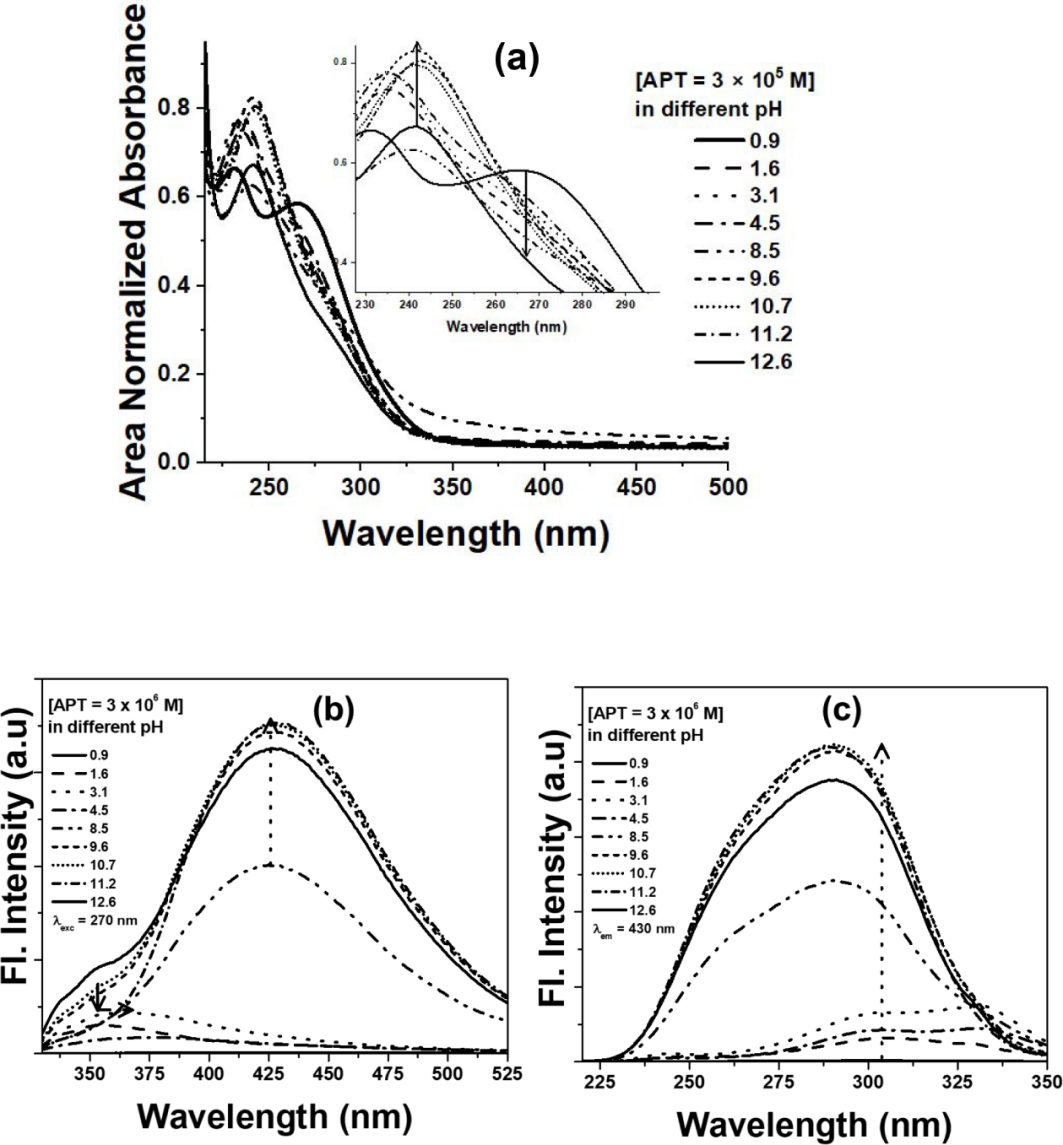
(a) UV–vis absorption, (b) emission (λ_exc_ = 270 nm), and (c) excitation (λ_em_ = 430 nm) spectra of APT at different pH levels. For UV–vis, the concentration of APT was maintained at 3 × 10^–5^ M, while that for fluorescence was 3 × 10^–6^ M.

In case of APT, the UV–vis spectra (Figure 6a) showed major absorbance changes under strongly acidic (pH < 3) and highly basic (pH > 12.6) conditions, caused by the protonation of triazole nitrogen or deprotonation of the aniline group. In contrast, spectra were stable between pH 4.5–10.7, consistent with a neutral, conjugated form. The fluorescence emission of APT (Figure 6b) peaked sharply at 400–430 nm, with maximum intensity in the mildly basic region (pH 8.5–11.2), where stabilization of the excited state enhances emission. Strong quenching occurred at very low and high pH, likely due to excited-state proton transfer or increased nonradiative decay pathways. Overall, APT displays dual-mode optical responsiveness, with optimal performance under mildly basic conditions, highlighting its potential as a pH-responsive fluorescent probe for sensing applications.

The spectroscopic response of ADT is influenced by the presence of amino groups due to the introduction of positive charges in proximity to the emissive chromophore.^31^ The emission maximum (Figure S1b) shifted toward low energy in a low pH environment. Variation of the pH did not seem to affect the UV–vis absorption (Figure S1a) of ADT. We thus presume that the protonation of the amine only influenced the molecule in the excited state following a typical charge-transfer emission.

### 2.4 DFT Studies

The photophysical properties of the triazole derivatives were confirmed via computational analysis of their molecular structures and their subsequent electron density distribution. Analyzing the optimized geometries of the molecules in differing media did not pose any significant alteration with respect to their skeletal structures in the ground state. DFT computations have well demonstrated the minimal effect of solvents on the overall core framework of molecules in transitioning between various media, though changes in other molecular parameters are prominent.^32,33^ In this context, the ground state optimized structures of (a) PTP, (b) NpTP, (c) PhTP, (d) ADT, and (e) APT in vacuum are shown in Figure 7. Although specific experimental details in the solvatochromic effects of NpTP were not reported in this study, we extended our investigation of the hydroxyaromatic framework by including computational analysis guided by previously published work on NpTP’s sensing behavior.^12^

**Figure 7:**
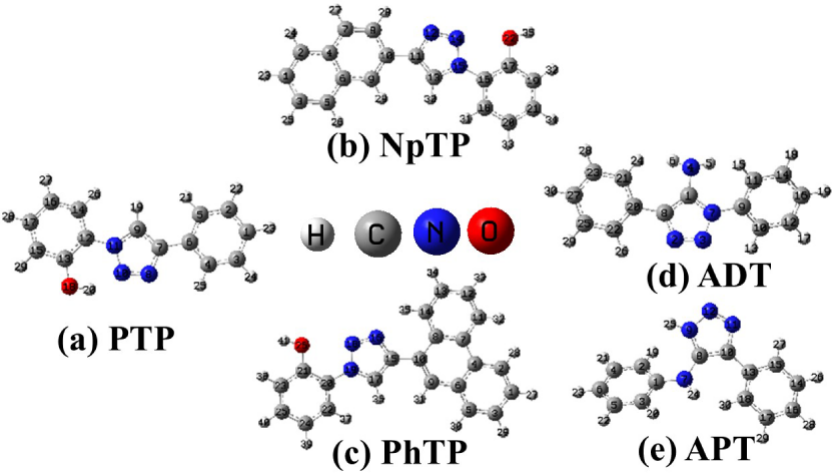
Ground-state optimized structures of (a) PTP, (b) NpTP, (c) PhTP, (d) ADT, and (e) APT in vacuum.

The excited-state optimized geometries of the compounds also revealed a similar skeletal characteristic feature (Figure S2). Depiction and analysis of their FMOs (Figure 8) in vacuum, water, methanol, and ACN in the ground state generated the corresponding FMO energy gaps in these media. The computed energy gaps enlightened us with the ease of reactivity of these molecules in different solvents. According to the FMO theory, the corresponding energy gap between its frontier molecular orbitals determines the feasibility of a reaction and selectivity. The smaller energy gap between their FMOs signifies a stronger reaction and better selectivity.^34,35^ From Figure 8, it is evident that the electronic charge distribution upon the molecules differed in their respective FMOs in the ground state. A closer screening of the FMOs in ADT and APT in vacuum revealed a more delocalized HOMO compared to the hydroxyaromatics, PTP, and PhTP in the ground state. PTP showed a significantly lower FMO energy gap even in the solvents compared to the others, suggesting greater electronic activity. The FMOs of PhTP and NpTP are more localized in solvents of increased polarity. NpTP has greater electron delocalization on the naphthalene ring and triazole moiety in its HOMO compared to the same on the hydroxy phenyl ring in its LUMO. The ground state HOMO–LUMO indicated a blue-shifted absorption of the hydroxyaromatic triazole, PTP, when the solvent changed from ACN to water, consistent with the trend in the experimental data. A similar trend is obvious in PhTP. Although a strong correlation in the trend for ADT with the experimental data was not observed, it is still relevant that methanol and ACN showed similar absorption maxima in both computational and experimental analysis. The theoretically computed data for APT in different solvents show a comprehensive correlation with the experimental data. The FMO diagrams may be represented as follows:

**Figure 8:**
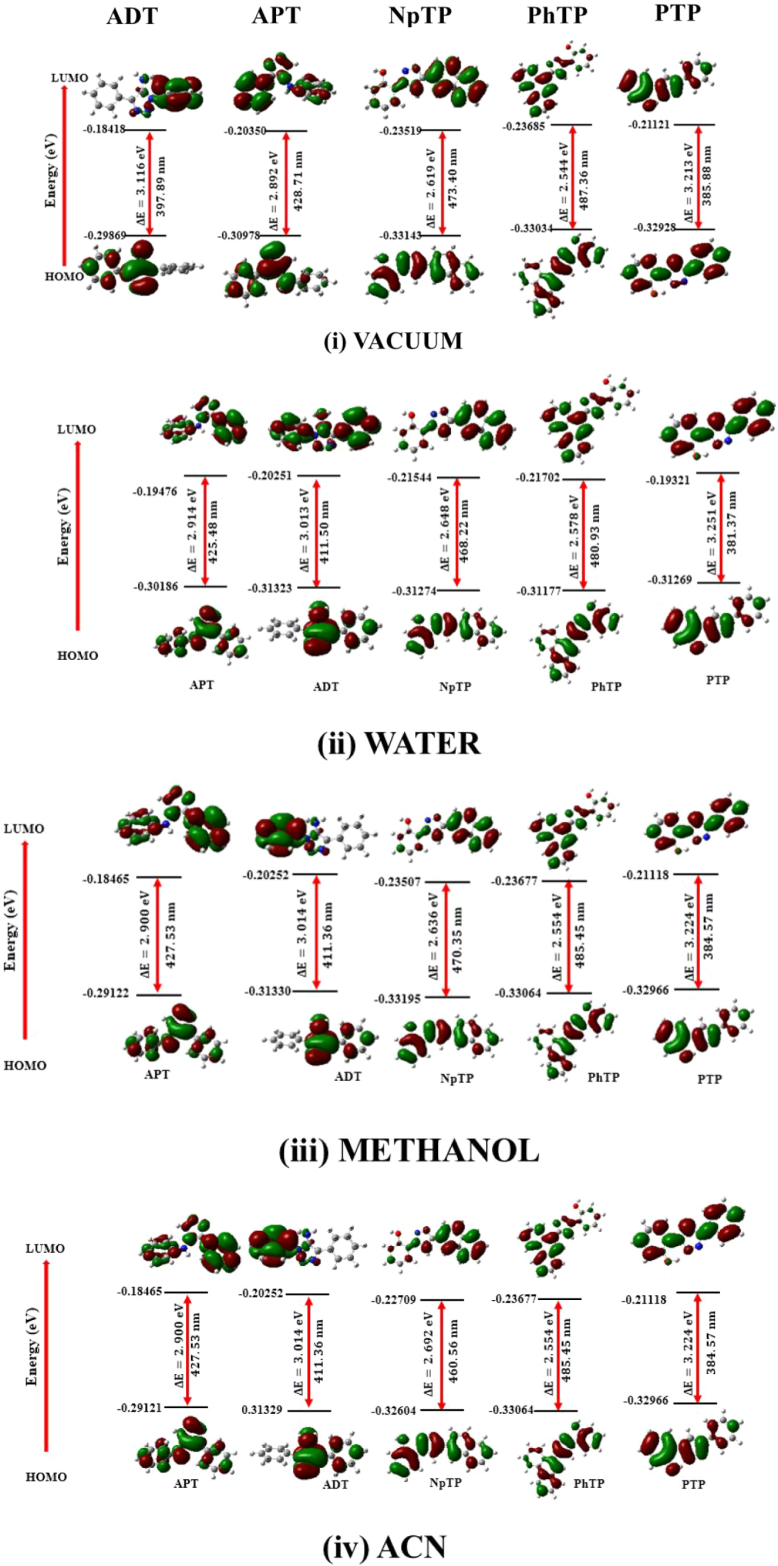
Ground-state HOMO–LUMO diagrams of the studied molecules in (i) vacuum and solvents of varying polarities (arranged in decreasing order of relative polarity index), (ii) water, (iii) methanol, and (iv) ACN, depicting the corresponding FMO energy gaps.

The excited state FMO characteristics (Figure 9) of the computed compounds in vacuum exhibited a relatively symmetric delocalization of electron density across their orbitals. With increasing polarity, the electron distribution on HOMO is centralized on the triazole and adjacent aryl groups for NpTP, PhTP, and PTP, whereas the same on LUMO is distributed on the hydroxy phenyl rings. These observations correlate with a previous computational study on PTP as a fluoride ion sensor.^9^ A strong intramolecular charge transfer character of the molecules in the excited state is signified, where the electron density is mainly transferred from the donor triazole moiety toward the acceptor hydroxy phenyl ring, as evident from Figure 9. In the gas phase, the highest FMO energy gap is observed for ADT with extensively localized electron density on the amino-substituted triazole moiety in HOMO. The electron density on LUMO is concentrated on the phenyl ring. This ultimately suggests that the electron-donating amino substituent enhanced the overall nucleophilicity of this system and stabilized the negative charges. In both methanol and ACN solvents, ADT revealed a similar electron density distribution, as clearly evident from its respective FMOs. This theoretical similarity may be accounted for by their comparable dielectric constants and refractive indices, leading to nearly equivalent solvent environments. ADT and APT also revealed an enhanced π-electron delocalization in the gas phase, indicating a strong charge transfer character in the absence of solvent interactions. In aqueous solvent (highly polar and protic), the excited state electron distribution in ADT and APT is extensively localized to maximize the charge separation between the donor and acceptor moieties, highlighting a pronounced charge transfer character. This becomes slightly reduced in low polar solvents, such as methanol and ACN.

**Figure 9:**
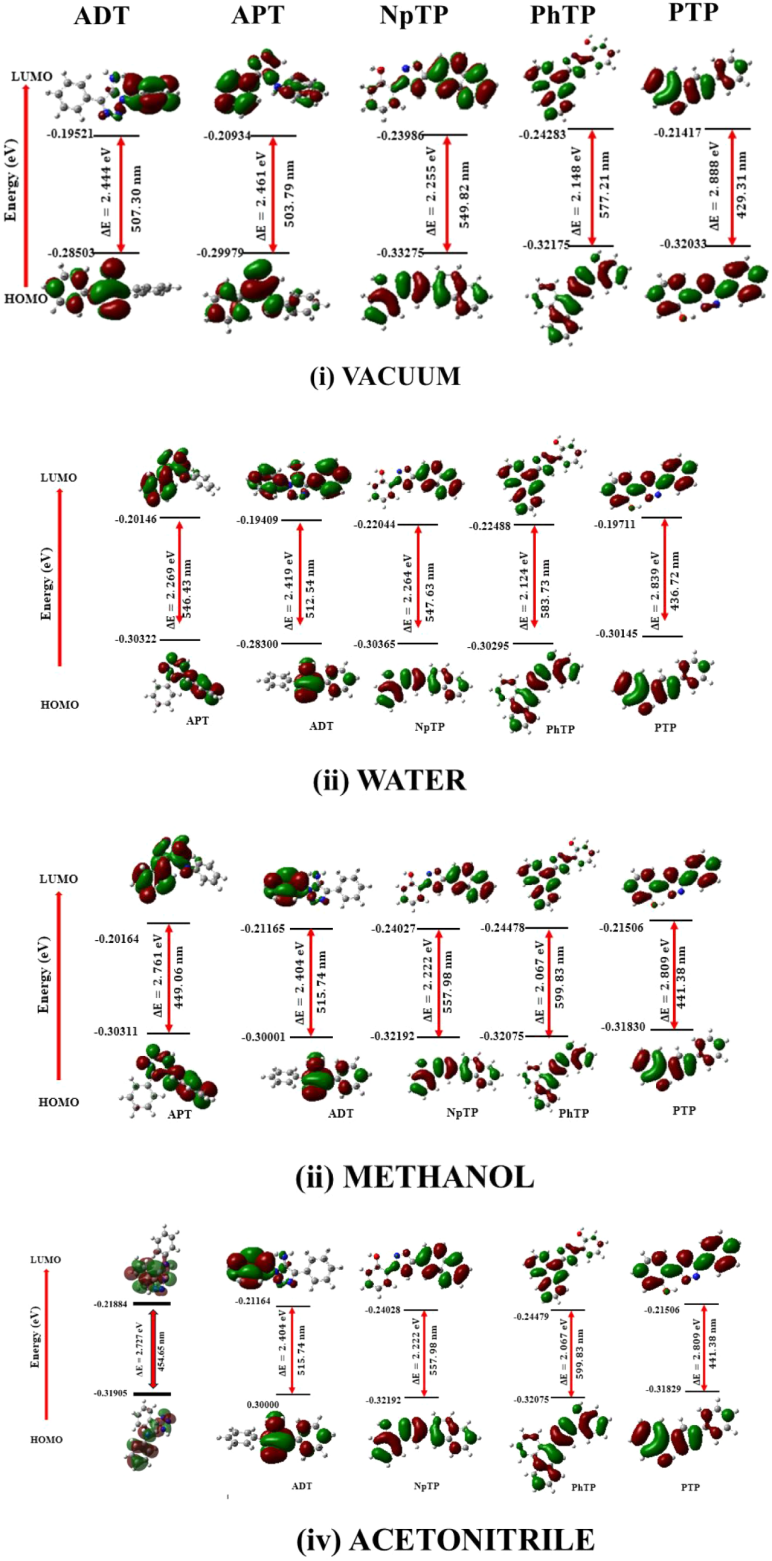
Excited-state HOMO–LUMO diagrams of the studied molecules (ADT, APT, NpTP, PhTP, PTP) in (i) vacuum and solvents of varying polarities (arranged in decreasing order of relative polarity index), (ii) water, (iii) methanol, and (iv) ACN, depicting the corresponding FMO energy gaps in the excited state.

A comparative representation of the observed and computed absorption (Table 1) and emission (Table 2) maxima has been tabulated for the solvent-specific characteristics of the 1,2,3-triazoles (PTP, PhTP, ADT, and APT). Despite discrepancies in the exact absorption maxima across solvents for the 1,2,3-triazoles, the observed and computed data exhibit a coherent and reproducible trend. The triazole molecules (PTP, PhTP, ADT, and APT) showed a red shift in the absorption spectra when the solvents were changed from water to a less polar medium. The emission maximum, however, illustrated a differential pattern for the hydroxyaromatic and nonhydroxyaromatic compounds. PTP and PhTP exhibited a slight, red-shifted emission from water to low-polarity solvents, as evident from the excited-state HOMO–LUMO. The nonhydroxyaromatic compounds, ADT and APT, however, depicted a blue-shifted emission in low-polarity solvents. This difference could be due to the inherent complex interactions between the functional group (−NH_2_) and the solvent, exhibiting solute–solvent specific interactions.

**Table 1:** Comparison of Experimentally Observed and Theoretically Computed Absorption Maxima of PTP, PhTP, ADT, and APT in Solvents of Varying Polarities

	Solvents					
	Water		Methanol		ACN	
1,2,3-Triazoles	λ_abs_^max^ (nm) (predicted)	λ_abs_^max^ (nm) (observed)	λ_abs_^max^ (nm) (predicted)	λ_abs_^max^ (nm) (observed)	λ_abs_^max^ (nm) (predicted)	λ_abs_^max^ (nm) (observed)
PTP	∼381	281.7	∼384	284.4	∼384	291.5
PhTP	∼481	292.0	∼485	298.04	∼485	300.0
ADT	∼412	260.1	∼411	266.9	∼411	268.9
APT	∼425	264	∼427	265	∼427	268

**Table 2:** Comparison of Experimentally Observed and Theoretically Computed Emission Maxima of PTP, PhTP, ADT, and APT in Solvents of Varying Polarities

	Solvents					
	Water		Methanol		ACN	
1,2,3-Triazoles	λ_em_^max^ (nm) (predicted)	λ_em_^max^ (nm) (observed)	λ_em_^max^ (nm) (predicted)	λ_em_^max^ (nm) (observed)	λ_em_^max^ (nm) (predicted)	λ_em_^max^ (nm) (observed)
PhTP	∼584	376.2	∼600	377	∼600	377.7
ADT	∼512	401.6	∼516	357.5	∼516	372.7
APT	∼546	435	∼449	420	∼427	390
PTP	∼437	380	∼441	403	∼441	-

#### 2.4.1 Electrostatic Surface Potential

DFT calculations provided clarity on the photophysical charge transfer characteristics of the molecules. This may be comprehensively portrayed through the electrostatic surface potential (ESP) mappings of these compounds in both ground and excited states in different media, as shown in Figures 10–14.

**Figure 10:**
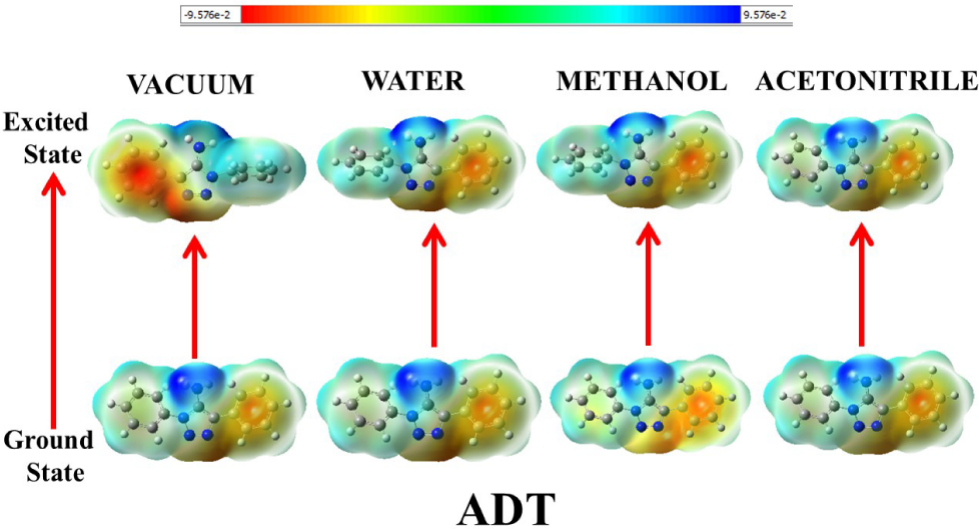
Ground- and excited-state ESP mappings of ADT in vacuum and solvents of varying polarities.

The ground state ESP of the nonhydroxyaromatic framework, ADT, in vacuum (Figure 10) depicted the molecule to be moderately polar with almost no intense positive or negative electron charge density spread over it. The excited state ESP, though, gave clear, intense red zones on the triazole ring, suggesting more negative charge accumulation due to its amino substituent, signaling toward electron redistribution on photoexcitation. The charge separations within the molecule, getting initiated in the excited states, are prominent, indicating the electrophilic and nucleophilic centers within the molecule. The difference in polarization between the amino-substituted triazole moiety and the phenyl rings is the prime factor for such charge separations, especially in a vacuum where there is no solvent polarization effective. In the ground state, the ESP variations of ADT are relatively consistent across solvents with increasing polarities. Intense charge separation in water signifies its high polar nature, followed by methanol and ACN. On the other hand, the excited state of ADT is stabilized through polarization as exposed through the high contrast of red and blue colors within it.^36^ The intramolecular charge transfer occurring within the aqueous solution of ADT in the excited state may be corroborated through this. This may be further explained through the excited state electrostatic potential map (EPM) in methanol, where the triazole and amino sites seem more involved in charge redistribution. In ACN, no significant charge distribution is noticed, and the molecule seems nonpolar. The charge redistribution in APT (Figure 11) also intensifies in the excited state compared to the ground state as revealed by its EPM. This marks a shift of electron density toward the triazoline moiety on excitation.

**Figure 11:**
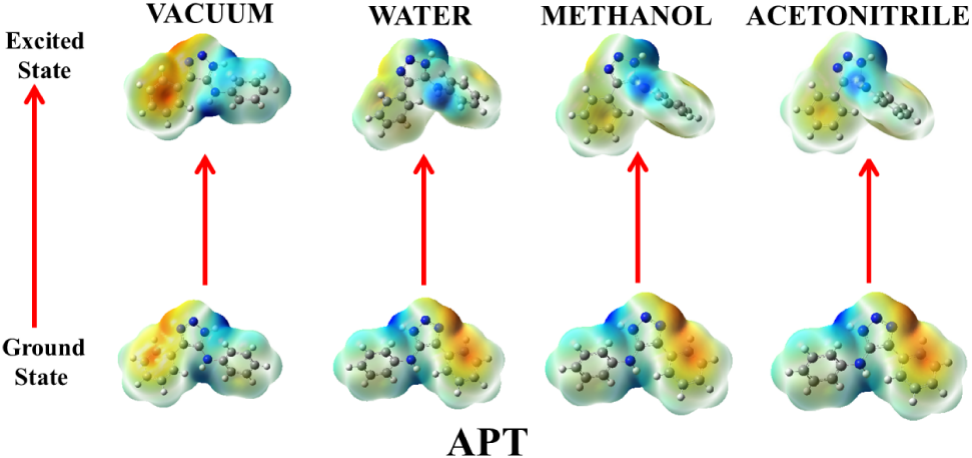
Ground- and excited-state ESP mappings of APT in vacuum and solvents of varying polarities.

NpTP and PhTP with the hydroxyaromatic skeleton presented a nearly nonpolar electrostatic potential mapping (Figures 12 and 13), especially in the ground state which indicates insignificant charge redistribution within these molecules. On the other hand, in aqueous solvent they show nearly similar ESP mapping comparable to ADT and APT in water. PhTP revealed quite intense charge separation between the phenanthrene—triazole ring and the hydroxy phenyl moiety as shown above. While NpTP and PhTP in MeOH showed darker ESP contrast in their excited state, and triazole and nearby locations light up more. There is no such pronounced solvent effect on NpTP and PhTP in ACN. Overall, ACN did not pull much charge, thereby reducing the ease of its charge transfer characteristics. Like the other triazole derivatives, PTP (Figure 14) also exhibited no significant difference in electron charge density between ground and excited states in vacuum. PTP, ADT and APT showed similar ESP in different solvents due to the presence of lesser conjugation and more prominent polarity because of their hydroxy and amino functionalities. Their excited state ESPs show strong color contrast, as evident from their strong charge transfer properties found experimentally.^37^

**Figure 12:**
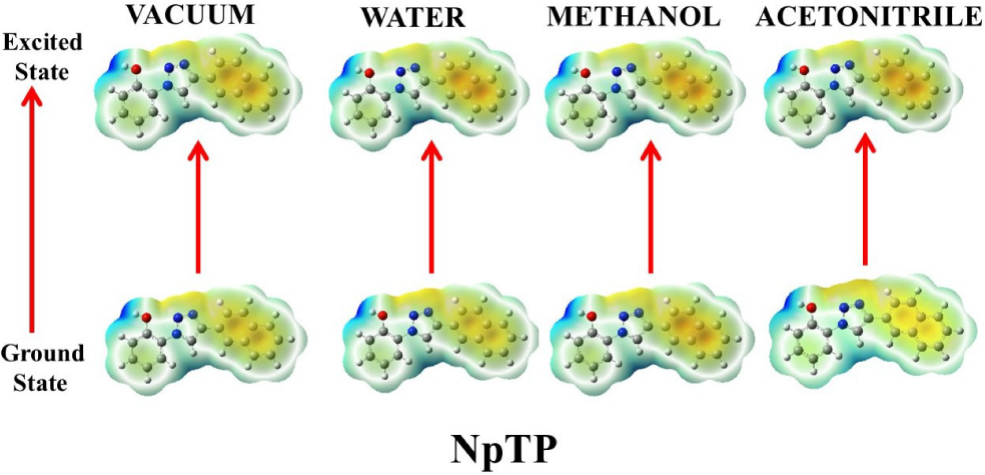
Ground- and excited-state ESP mappings of NpTP in vacuum and solvents of varying polarities.

**Figure 13:**
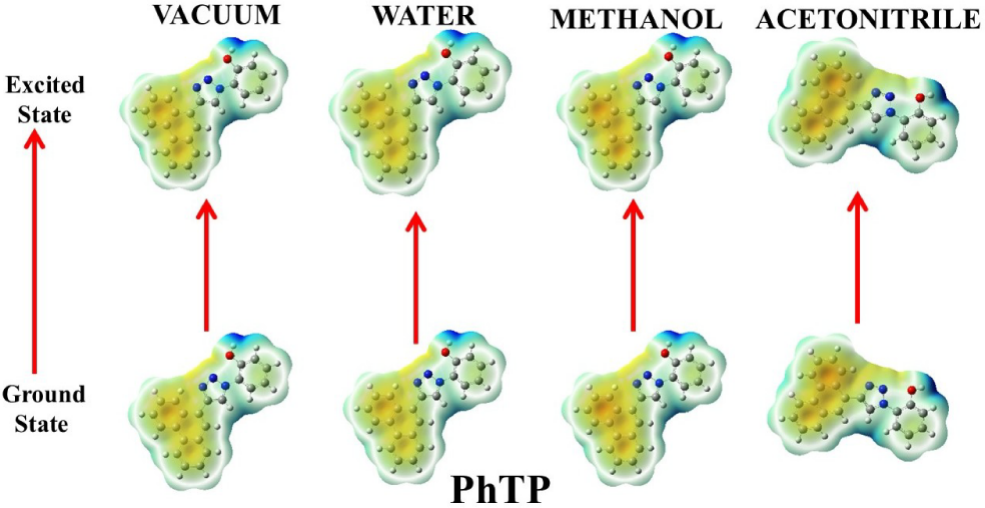
Ground- and excited-state ESP mappings of PhTP in vacuum and solvents of varying polarities.

**Figure 14:**
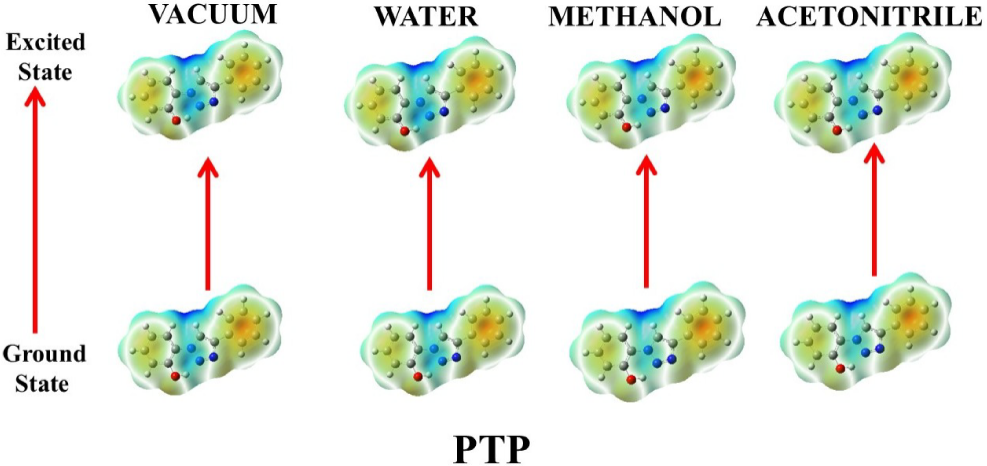
Ground- and excited-state ESP mappings of PTP in vacuum and solvents of varying polarities.

The Mulliken charges extracted for the studied compounds explicitly explained the shift in various environments (*vide* Tables S1–S5 in Supporting Information). Analyzing the Mulliken charges for the hydroxyaromatic triazole derivatives PTP, NpTP, and PhTP revealed consistent charge distribution across the different electronic environments within the parent PTP framework. The oxygen atom of the phenolic −OH group in PTP retained a large negative charge, confirming its hydrogen donor/acceptor site. NpTP and PhTP presented slightly less negative charge on the “O” atom and slightly less positive charge on the “H” atom compared to PTP. This hinted at modest charge delocalization into the larger aromatic substituents (naphthalene and phenanthrene). The triazole nitrogen atoms in PTP, NpTP, and PhTP present unique electron charge characteristics. The N8 and N11 atoms in PTP are distinctly negative, whereas the N10 atom is slightly positive in the ground state. In the excited state, the positivity increases with the increase in solvent polarity, reinforcing the intramolecular charge transfer characteristics. Owing to their increased conjugation, NpTP and PhTP showed more negative N10 and N11 atoms. On the other hand, the N10 atom revealed a greater inclination toward positive charge in the excited state or with increasing solvent polarity, signifying stronger ICT from the phenolic end to the aromatic substituent via the triazole bridge. The carbon atoms also exhibited similar characteristics with the triazole-bound carbon (C13) in PTP being more positively charged in vacuum/water and then shifting toward the negative end in methanol/ACN. The increased conjugation/resonance effect on the naphthalene and phenanthrene rings of NpTP and PhTP makes the C13 charge magnitude moderate, with smaller polarity swings across solvents. A closer scrutiny of the aromatic C—H hydrogens revealed them to remain largely unaffected by the substituent size. The excited state analysis disclosed a rather consistent Mulliken charge redistribution with increasing conjugation: PTP, NpTP, and PhTP, especially within the polarized solvent environments, which corroborate their charge transfer characteristics.

In the ground state, gas phase, the nitrogen atom of the amino group (N4) in ADT is profoundly negative, indicating its strong electron-rich character, whereas the C1 atom of the phenyl ring is positive, thus, the propensity of electron donation is expected from the phenyl ring toward the triazole core. These extreme charge separations are moderated by the introduction of solvents, though the characteristics remain similar. The positivity at C1 increases on excitation and slightly intensifies the negative Mulliken charge at N4, suggesting intramolecular charge transfer (ICT) from phenyl ends to the amino-triazole core. APT in its ground state reveals the same characteristic in a vacuum. The aniline substituent shows significant electron donation into the triazole system on excitation. The C13 atom in APT becomes highly electron-deficient in the excited state, signaling a strong charge-transfer to occur from aniline to the triazole core.

#### 2.4.2 Natural Bond Orbital (NBO) Analysis

Natural Bond Orbital (NBO) analysis provides comprehensive insights into the intra- and intermolecular orbital interactions in a molecule that are prospective candidates for charge–transfer transitions. NBO analysis of molecules containing filled donor and empty acceptor orbitals gives us an appropriate foundation to theoretically investigate the propensity of charge-transfer transitions or conjugative interactions in such molecular systems.^38^ Second-order perturbation energies are useful metrics to delve into the origin of molecular stabilization by analyzing interactions between Lewis NBOs (bond pairs and lone pairs) as “donors” and non-Lewis NBOs (antibonding or Rydberg) as “acceptors”.^39^ Second-order perturbation energies are computed as given by Equation 1:

1 $$E\left(2\right)=-q_{i}F_{ij}/\left(\epsilon_{i}-\epsilon_{j}\right)$$

where *q_i_* is the population of the donor orbital; ε_*i*_, ε_*j*_ are the orbital energies of donor and acceptor NBOs, respectively; and *F_ij_* is the off-diagonal Fock or Kohn–Sham matrix element between *i* and *j* NBOs.

The donor–acceptor interactions of APT (hydroxy aromatic) and PTP (non–hydroxy aromatic) in solvent water are depicted in Tables 3 and 4, respectively.

**Table 3:** Major Donor–Acceptor Interactions of PTP in Water (GS) and Their Second-Order Perturbation Energies (kcal/mol)^a^

Donor NBO (*i*)	Acceptor NBO (*j*)	*E*(2) (kcal/mol)	Δ*E* (a.u.)	*F*(*i*, *j*) (a.u.)
BD (2) C_1_–C_3_	BD* (2) C_2_–C_5_	20.12	0.28	0.067
BD (2) C_1_–C_3_	BD* (2) C_4_–C_6_	20.58	0.28	0.068
BD (2) C_2_–C_5_	BD* (2) C_1_–C_3_	19.64	0.28	0.066
BD (2) C_2_–C_5_	BD* (2) C_4_–C_6_	19.83	0.28	0.067
BD (2) C_4_–C_6_	BD* (2) C_1_–C_3_	19.96	0.28	0.067
BD (2) C_4_–C_6_	BD* (2) C_2_–C_5_	19.59	0.28	0.067
BD (2) C_4_–C_6_	BD* (2) C_7_–C_9_	19.04	0.26	0.062
BD (2) C_7_–C_9_	BD* (2) N_8_–N_10_	29.71	0.21	0.076
BD (2) N_11_–C_12_	BD* (2) C_7_–C_9_	26.64	0.08	0.052
BD (2) N_11_–C_12_	BD* (2) C_14_–C_16_	71.67	0.10	0.094
LP (1) C_13_	BD* (2) N_11_–C_12_	339.84	0.05	0.124
LP (1) C_13_	BD* (2) C_14_–C_16_	53.6	0.16	0.102
LP (2) O_18_	LP (1) C_13_	58.96	0.19	0.123

a The labeled GS geometry-optimized structure of PTP is provided in Figure 5 for easy correlation [BD = bonding orbitals; LP = lone pair; CR = core orbitals; BD* = antibonding orbitals].

**Table 4:** Major Donor–Acceptor Interactions of APT in Water (GS) and Their Second-Order Perturbation Energies (kcal/mol)^a^

Donor NBO (i)	Acceptor NBO (j)	*E*(2)(kcal/mol)	Δ*E* (a.u.)	*F*(*i*, *j*) (a.u.)
BD (2) C_1_–C_2_	BD* (2) C_4_–C_6_	21.46	0.29	0.071
BD (2) C_1_–C_2_	BD* (2) C_3_–C_5_	17.12	0.29	0.063
BD (1) C_2_–C_4_	BD* (1) C_1_–N_7_	4.46	1.12	0.063
BD (2) C_1_–C_2_	BD* (2) C_4_–C_6_	256.43	0.01	0.081
BD (2) C_4_–C_6_	BD* (2) C_1_–C_2_	18.63	0.27	0.064
BD (2) C_3_–C_5_	BD* (2) C_1_–C_2_	21.55	0.27	0.07
BD (2) C_4_–C_6_	BD* (2) C_3_–C_5_	22.24	0.28	0.07
BD (2) C_8_–C_10_	BD* (2) C_13_–C_18_	92.76	0.02	0.061
BD (2) C_8_–C_10_	BD* (2) N_11_–N_12_	31.11	0.22	0.079
LP (1) N_7_	BD* (2) C_1_–C_2_	28.72	0.29	0.084
LP (1) N_7_	BD* (2) C_8_–C_10_	35.31	0.28	0.093
LP (1) N_9_	BD* (2) C_8_–C_10_	42.91	0.29	0.101
LP (1) N_9_	BD* (2) N_11_–N_12_	40.91	0.24	0.089

a The labeled GS geometry-optimized structure of APT is provided in Figure 5 for easy correlation [BD = bonding orbitals; LP = lone pair; CR = core orbitals; BD* = antibonding orbitals].

The above table indicates several donor–acceptor interactions in the ground state of PTP in water. The energies ∼19–21 kcal/mol denote normal π → π delocalization extending within the phenyl rings across the triazole linker. This strong delocalization energy is responsible for the stability of PTP. Significant π → π* interactions, among π (C_7_–C_9_) → π* (N_8_–N_10_); π (N_11_–C_12_) → π* (C_7_–C_9_); π (N_11_–C_12_) → π* (C_14_–C_16_) between the triazole moiety and the phenyl rings shows strong electronic coupling with strong perturbation energies 26–72 kcal/mol. Further, LP (2) O_18_ conjugates with π* C_13_ with a stabilization energy of 58.96 kcal/mol. The largest delocalization energy ∼340 kcal/mol implies strong LP (2) → π* (N_11_–C_12_) donation.

A similar NBO analysis was performed for the molecule APT in its ground state in water. Typical π → π delocalization within the phenyl rings and adjacent C–C bonds is suggested by the second perturbation energies 17–22 kcal/mol. A strong cross–linking between the phenyl rings and the triazole moiety is suggested through the π (C_8_–C_10_) → π* (C_13_–C_18_); π (C_1_–C_2_) → π* (C_4_–C_6_) interactions. LP (1) N_7_ → π (C_8_–C_10_) and LP (1) N_9_ → π (C_8_–C_10_)/(N_11_–N_12_) interactions with second-order perturbation energies of 29–43 kcal/mol indicate the participation of the nitrogen lone pairs of the triazole moiety, stabilizing the conjugated system.

#### 2.4.3 Dipole Moment

Theoretical derivation of ground and excited state dipole moments of the computed molecules often quantitatively supports their photophysical characteristics and solvatochromic behavior as explained from their FMO and EPM analyses. In the present case, the computed ground and excited state dipole moments of PTP, NpTP, PhTP, ADT, and APT are tabulated (Table 5).

**Table 5:** Computed Ground and Excited State Dipole Moments of the 1,2,3-Triazoles

		Dipole moment in different environments (μ) Debye							
		Vacuum		Water		Methanol		ACN	
Sl. no.	Compounds	GS (μ_g_)	ES (μ_e_)	GS (μ_g_)	ES (μ_e_)	GS (μ_g_)	ES (μ_e_)	GS (μ_g_)	ES (μ_e_)
1	PTP	4.15	5.07	6.20	6.01	6.12	5.93	6.13	5.95
2	NpTP	4.59	6.28	6.75	4.61	6.66	4.51	6.68	4.53
3	PhTP	4.79	5.91	6.93	5.04	6.84	4.84	6.85	4.87
4	ADT	5.21	13.99	7.82	17.85	7.72	17.74	7.74	17.75
5	APT	5.05	11.04	7.15	17.11	7.08	16.96	7.09	7.16

The dipole moment data aided in the interpretation of the structure–activity insights for the compounds studied. The hydroxyaromatic triazoles, PTP, NpTP, and PhTP revealed moderate (μ_g_) ∼4.15–4.79 D in vacuum, which significantly increased to ∼6.2–6.9 D in solvents. This enhancement is attributed to the electron-donating phenolic −OH group and the extended π-conjugation across the aromatic system in conjunction with the triazole ring.

The dipolar orientation of the amino-substituted triazole in ADT resulted in the highest gas-phase dipole moment (μ_g_) in vacuum among the series. This value further increased to approximately ∼7.7 D in solvent environments, reflecting enhanced polarization due to solute–solvent interactions and the electron-donating nature of the −NH_2_ group. APT in vacuum (5.04 D) showed a similar value to ADT due to the anilino functionality, and the same in solvents is enhanced like ADT. A significant increase in the excited state dipole moments (μ_e_) in all the compounds points toward the redistribution of the intramolecular charge on subsequent excitation, a common characteristic in π donor–acceptor systems. The variation in the trends of dipole moment values of the studied compounds in the excited state is in close alignment with the ground state. Overall, the polarity effect induced by the solvents in the hydroxyaromatics (PTP, NpTP, and PhTP) on excitation is somewhat reduced in comparison to ADT and APT owing to their (- NH_2_) and (- NH-Ph) substitutions. Similarity in such observation was analyzed from the ESP characteristics of these molecules, where the ICT characteristics in ADT and APT were more pronounced than the hydroxy aromatics.

## 3 Experimental Section

### 3.1 Materials and Methods

All chemicals and reactants were obtained from commercial sources and used without further purification. The 1,2,3-triazoles with the hydroxyaromatic skeletal framework, 2-(4-phenyl-1H 1,2,3-triazol-1-yl) phenol (PTP), 2-(4-(naphthalen-2-yl)-1H-1,2,3-triazol-1-yl) phenol (NpTP) and (2-[4-(phenanthren-9-yl)-1H-1,2,3-triazol-1-yl]­phenol (PhTP) were synthesized and reported in previous publications.^10,12,13^ The other triazoles, 5-amino-1,4-diphenyl-1,2,3-triazole (ADT) and 5-anilino-4-phenyl-1H-1,2,3-triazole (APT), were purchased from Sigma-Aldrich. The solvents, such as hexane (Hex), heptane (Hep), ethanol (EtOH), methanol (MeOH), and acetonitrile (ACN), obtained from various sources (Fisher Scientific, Sigma-Aldrich, etc.), were used after drying with molecular sieves. Molecular sieves can reduce the water content in organic solvents to sub-10 ppm levels.^40^ Glycerol (≥99.0%) was purchased from Sigma-Aldrich and used as received. Deionized water (pH 6.5–7.3) was used as another solvent.

### 3.2 Preparation of 1,2,3-Triazoles in Different Solvents

A concentrated stock (∼1 × 10^–3^ M) of the individual triazole compounds was prepared in various solvents. PTP and PhTP were tested in hexane, ACN, methanol, ethanol, and water. ADT was studied in ACN, methanol, and water, while APT was tested in heptane, ACN, methanol, ethanol, and water. A calculated amount from the stock solution was then added to different vials with the specific solvents, resulting in the concentration of PTP as 2 × 10^–5^ M, NpTP and PhTP as 1 × 10^–5^ M, ADT and APT as 2 × 10^–6^ M in the cuvettes for spectroscopic analysis. The absorption linearity of APT has been represented in Figure S4. This range of concentration avoided any possible intermolecular effect. The final solutions were thoroughly mixed with a vortex mixer (rpm ∼ 1000) before testing. The wet-lab experiments with PhTP were reproduced from earlier studies as part of the present solvent investigation.^20^ The results were reproduced based on the procedures from recently published articles, allowing for a direct comparison with the other 1,2,3-triazole derivatives.

### 3.3 Calculation of Quantum Yield

For the evaluation of quantum yield, the 1,2,3-triazole molecules were individually dissolved in the respective solvents. The solutions were diluted to maintain an absorbance of ∼0.3 to obtain an absorption profile, and the optical density at $\lambda_{\max}^{\mathrm{abs}}$ . These solutions were further diluted (nearly 10 times) to obtain fluorescence spectra to collect the integrated emission intensity (Area under fluorescence). Quantum yield of the sample (Φ_S_) was calculated using quinine sulfate as the standard. The quantum yield of quinine sulfate, 0.546, as reported in 0.1 M H_2_SO_4_, was the reference quantum yield (Φ_ref_) at an excitation maximum of 340 nm.^41^ To correlate the excitation maxima of the individual triazoles (270–330 nm), quinine sulfate was excited at 270 nm (Φ_ref_ at 270 nm = 0.43 ± 0.03) and 310 (Φ_ref_ at 310 nm = 0.50 ± 0.04) nm to assess the quantum yield at these maximum excitation wavelengths, which was used as the reference for the triazoles. Equation 2 was used to calculate the sample quantum yields

2 $$\Phi_{S}=\Phi_{\mathrm{ref}}\cdot \frac{{\left(\mathrm{Area under fluorescence}\right)}_{S}}{{\left(\mathrm{Area under fluorescence}\right)}_{\mathrm{ref}}}\cdot \frac{{\left(\mathrm{Absorbance}\right)}_{\mathrm{ref}}}{{\left(\mathrm{Absorbance}\right)}_{S}}\cdot \frac{\eta_{S}^{2}}{\eta_{\mathrm{ref}}^{2}}$$

where η_S_ and η_ref_ are the refractive indices of the sample and reference, respectively.

### 3.4 Characterization Techniques

Room temperature UV–vis absorption was conducted using a Shimadzu UV-2600i spectrophotometer, while steady-state fluorescence measurements were performed using PerkinElmer LS55, and Horiba Scientific FluoroMax Plus fluorimeters. The UV–vis spectrometer is a double-beam system with a scan range of 190–900 nm (wavelength accuracy ± 0.1 nm @ 656.1 nm D2 and +0.3 nm all range) and *R*-928 photomultiplier tube. For the experiment, the scan speed was kept at medium, and the slit width was 2 nm. The solvent subtraction was conducted by using the baseline correction technique. A UV–vis scan of the solvent was taken to recheck solvent correction. For the fluorescence experiments, the scan range depended on the excitation and emission wavelengths monitored. The excitation and emission monochromator slits were kept at 2.5 nm for all experiments, and the integration time was 0.2 s. A blank spectra of the solvent for each excitation wavelength was collected to assess the quality of the solvents. The fluorometric analyses were conducted using a combination of FluorEssence software from Horiba and MS Excel after importing the ASCII files from the PerkinElmer LS55 instrument.

### 3.5 Computational Methods

A widely adopted technique to investigate the structure–activity correlation of a molecule invokes theoretical simulation deploying density functional theory (DFT). This high-throughput technique provided comprehensive clarity encompassing the structural characteristics of many-electron systems along with their electronic behavior, facilitating deeper insights into their functional characteristics.^42,43^ Using the computational chemistry software Gaussian 09W, we generated the stable ground state electronic structures of the triazole derivatives PTP, NpTP, PhTP, ADT, and APT through full optimization of their geometrical parameters, such as bond length, bond angle, and dihedral angle.^44^ DFT was employed to optimize the systems using the B3LYP functional and 6-311++G** basis set, which offered a good compromise between computational accuracy and cost. All computations were performed under gas-phase and solvent conditions. Solvents (water, methanol, and ACN) were employed implicitly using the Polarizable Continuum Model (PCM) to mimic solvation effects by representing the solvent as a continuous polarizable dielectric medium.^45,46^ The excited state geometries and the absorption and emission energies were calculated using the TD-DFT (Time-Dependent DFT) approach, applying the same technical methodology.^47,48^ The default FineGrid integration grid was employed for all the DFT studies. All DFT computations, ground and excited state geometry optimizations, and SCF procedures converged with tight convergence criteria (energy change ∼1 × 10^–6^ A.U. and maximum density matrix threshold ∼1 × 10^–8^). Spin contamination checks were not performed in unrestricted calculations, and slight spin contamination is hereby acknowledged. The B3LYP/6-311++G** protocol tagged with PCM though, is a less advanced functional compared to the modern CAM-B3LYP or ωB97X-D or hybrid methods for computing charge transfer excited states. Still, its usage is convenient and effective in certain aspects like cost-effectivity, computational robustness, etc. Sandoval et al.^49^ in their article revealed the B3LYP functional to be more effective than any other advanced functional, like CAM-B3LYP. In the articles of Nayyar et al.^50^ and Wobbe et al.,^51^ the B3LYP functional outperformed CAM-B3LYP or other hybrid functionals to draw a correlation between experimental and theoretical results.

The Frontier Molecular Orbitals (FMOs) were generated from the optimized/computed chk.point files of the computed molecular structures of the compounds and visualized through the GaussView 5.0 software. Electronic indices include the energies of the lowest unoccupied molecular orbital (*E*_LUMO_) and the highest occupied molecular orbital (*E*_HOMO_) of the triazole derivatives.^52,53^ The distribution of electronic charges, oscillator strengths, electronic transition energies, electrostatic potential (ESP) maps dipole moment, and thermodynamic properties of PTP, NpTP, PhTP, ADT and APT in differing media are also computed and obtained through DFT analysis via Gaussian 09W. Natural bond orbital (NBO) analysis of representative hydroxy aromatic, APT and nonhydroxy aromatic, PTP are performed in water for their ground state optimized structures.^54^

## 4 Conclusion

In summary, this comprehensive study presented a structure–property investigation of a series of disubstituted 1,2,3-triazoles, integrating steady-state UV–Vis and fluorescence spectroscopy with density functional theory (DFT) calculations to decipher how subtle structural modifications modulate the electronic and photophysical behaviors of the compounds. The hydroxyaromatic triazoles (PTP, NpTP, and PhTP) demonstrated characteristic π–π* transitions and moderate solvatochromism, while their emission profiles reflected the influence of extended conjugation and solvent polarity. Among them, PhTP emerged as the most fluorescent due to its rigid polyaromatic substitution. In contrast, the nonhydroxyaromatic triazoles (ADT and APT), although isomeric, displayed starkly different photophysical behavior, attributed to differences in electron-donating group orientation and charge-transfer propensity. APT, in particular, showed remarkable solvent-sensitive emission with high quantum yields in aprotic media, underscoring its potential as an environment-responsive fluorophore. pH studies indicated that the photophysical properties of 1,2,3-triazoles are governed by site-specific protonation and deprotonation equilibria. PTP and PhTP exhibit spectral changes linked to phenolic −OH groups, while APT showed dual-mode responsiveness with optimal emission under mildly basic conditions. ADT, on the other hand, displayed primarily excited-state effects due to amine protonation.

DFT calculations corroborated the aforementioned experimental observations by revealing marked variations in HOMO–LUMO electron density distributions, computed energy gaps between the HOMO and LUMO, electrostatic surface potentials, and dipole moments in across media. Additionally, the solvatochromic effects on these molecules were theoretically justified through computations and analysis in the gas phase and across solvents of varying polarities. These computational insights also augmented the effects of subtle structural modifications on the electronic properties of these molecules. The core triazole framework remains geometrically consistent, and the electronic environments are highly tunable with substituent identity and solvent polarity. The observed intramolecular charge transfer (ICT) characteristics, particularly in ADT and APT, were reinforced by excited-state analyses, in comparison to PTP, NpTP, and PhTP, establishing a firm link between molecular architecture and emissive behavior.

In a nutshell, this work demonstrated that the photophysical landscape of triazole-based systems can be finely controlled through rational structural design and environmental modulation. The insights gained from this study offer valuable guidelines for engineering new triazole derivatives for sensing, imaging, and optoelectronic applications.

## Supplementary Material



## Notes

A comprehensive photophysical analysis of 1,2,3-triazole derivatives and related compounds (PTP, PhTP, ADT, APT) across varied solvents and pH conditions; it includes spectral characterization (absorption, emission, and excitation) under different pH and solvent environments; excited-state structural optimization of triazoles; polarity estimation across media; fluorescence deconvolution and quantum yield data; and concentration-dependent and intensity vs time-related emission studies (PDF)
