# Supplementary material for: Structure–Property Correlations in Disubstituted 1,2,3-Triazoles: DFT Insights and Photophysical Analysis
Source: ACS Omega. 2025 Nov 24;10(48):58925–42. doi: 10.1021/acsomega.5c07623 (PMC12771426; doi:10.1021/acsomega.5c07623)
Supplement: Supplementary file 1 [file ao5c07623_si_001.pdf]

Structure – Property Correlations in Disubstituted 1,2,3-Triazoles: DFT Insights and Photophysical Analysis

Dharatiben Lakhani[a], Sheeba Sadiq[a], Harini Subbaiahgari[a], Violet Swanson[a], Jacob Munyon[a], Sher B. Poudel[a], Karelle S. Aiken[b], Shainaz M. Landge[b], Debosreeta Bose*[c], Debanjana Ghosh*[a]

[a]Department of Chemistry, Science Building West, Box - 1652, Southern Illinois University Edwardsville, Edwardsville, IL 62026-1652, USA.

[b]Department of Biochemistry, Chemistry, and Physics, Georgia Southern University (Statesboro Campus), 521 College of Education Drive, Statesboro, GA 30460-8064, USA.

[c]Department of Chemistry, Amity Institute of Applied Sciences (AIAS), Amity University - Kolkata Campus, Rajarhat, Newtown, Kolkata, West Bengal, 700135, India.

E-mail: *[a]dghosh@siue.edu ; *[c]dbose@kol.amity.edu

Supporting Information

Table of Contents

**Contents**	**Pages**
Absorption and emission spectra of ADT at different pH	S2
Excited state optimized structures of the 1,2,3-triazoles	S3
Estimation of polarity of the 1,2,3-triazoles in different media	S4-S11
Deconvoluted fluorescence emission spectrum of PhTP in ACN	S12
Table of absorption and emission maxima, Stokes shifts, and Quantum yield of 1,2,3-triazoles	S13-S14
Concentration-dependent controls of APT in ACN	S15
pH variation studies of PTP, PhTP, and APT	S17-S19
Excitation and emission scans of 1,2,3-triazoles in ACN	S20
Excitation and emission scans of PhTP in hexane and ethanol	S21
Absorption, emission, and excitation scans of APT in glycerol-water mixture	S22
Emission intensity vs. time of PhTP	S23

Absorption and emission spectra of ADT at different pH

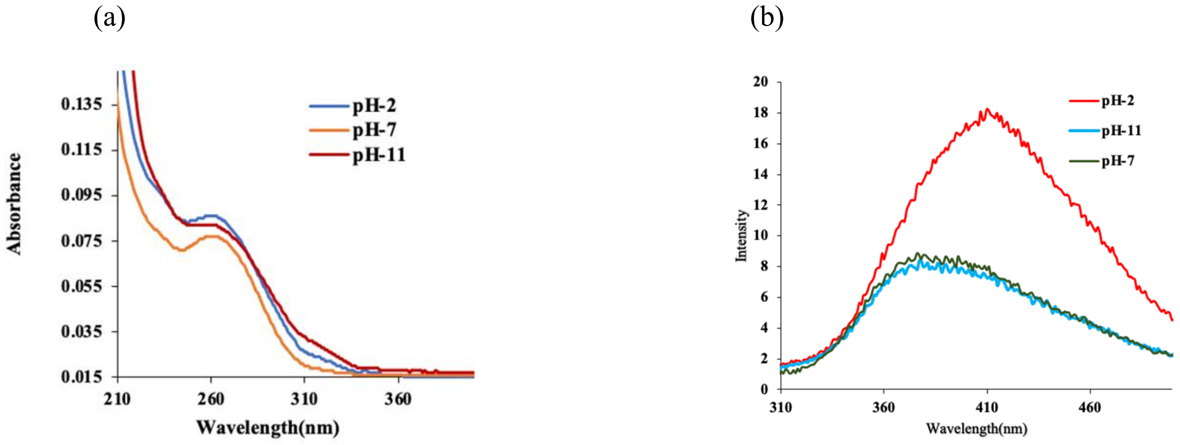

Figure S1. (a) UV-Vis absorption spectra, and (b) emission scans (λ_{exc} = 270 nm) of **ADT** in different pH.

Excited state optimized structures of the 1,2,3-triazoles

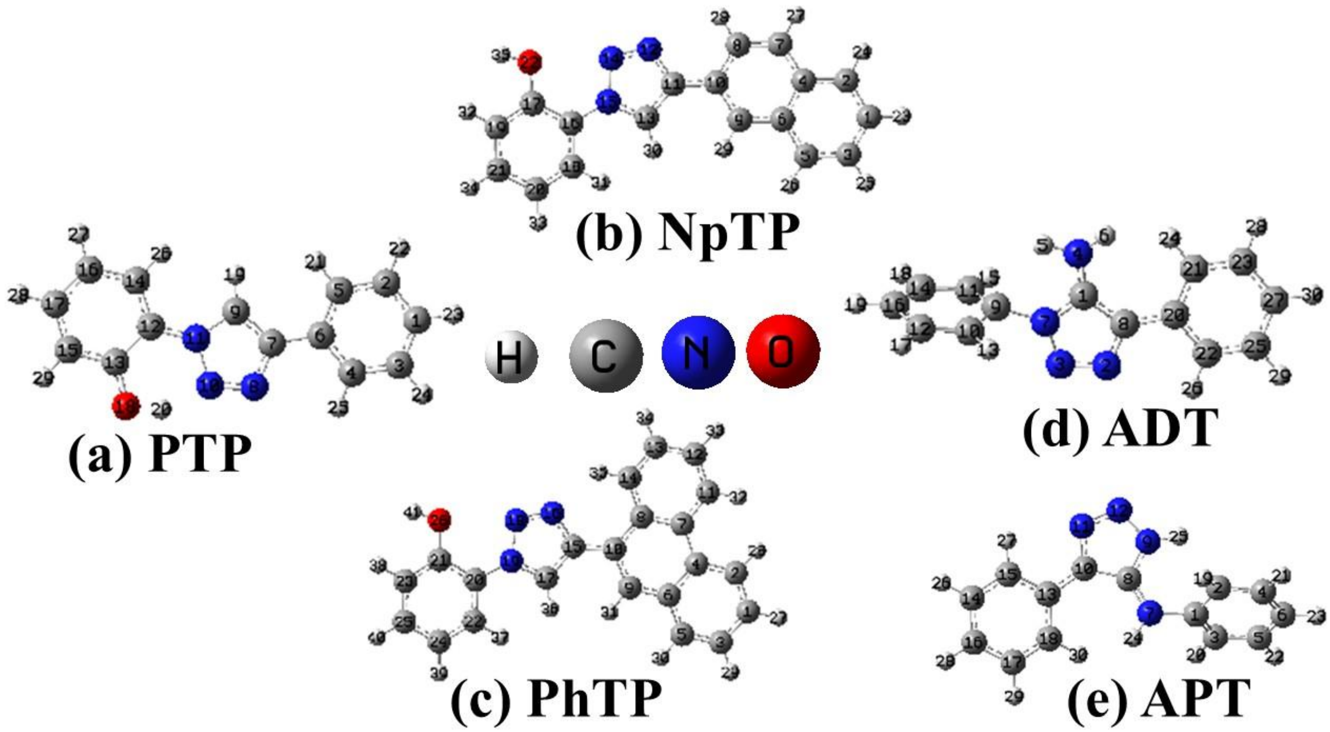

Figure S2. The excited state optimized structures of (a) **ADT** (b) **NpTP** (c) **PhTP** (d) **PTP** (e) **APT** in vacuum.

Table S1. Estimation of polarity of **ADT** in Vacuum and in solvents of varying polarities

**ADT**	VACUUM		WATER		METHANOL		ACETONITRILE	
Atom	Ground state	Excited state	Ground state	Excited state	Ground state	Excited state	Ground state	Excited state
**C1**	0.764	0.853	0.769	0.849	-0.691	-0.273	-0.691	-0.274
**N2**	-0.484	-0.489	-0.494	-0.506	-0.119	-0.028	-0.119	-0.028
**N3**	0.038	0.015	0.029	0.004	0.111	0.083	0.011	0.085
**N4**	-1.106	-1.155	-1.118	-1.159	-0.679	-0.691	-0.679	-0.690
**H5**	0.456	0.473	0.457	0.472	0.461	0.461	0.461	0.461
**H6**	0.455	0.470	0.456	0.471	0.475	0.499	0.475	0.499
**N7**	-0.394	-0.367	-0.388	-0.369	0.429	0.470	0.429	0.470
**C8**	0.107	0.134	0.111	0.133	0.297	0.104	0.298	0.104
**C9**	0.424	0.371	0.401	0.378	-1.025	-1.352	-1.025	-1.352
**C10**	-0.418	-0.398	-0.406	-0.401	-0.336	-0.008	-0.336	-0.009
**C11**	-0.418	-0.396	-0.408	-0.394	0.392	0.167	0.393	0.168
**C12**	-0.390	-0.413	-0.390	-0.410	-0.172	-0.373	-0.172	-0.374
**H13**	0.380	0.383	0.382	0.383	0.290	0.295	0.290	0.295
**C14**	-0.390	-0.414	-0.390	-0.412	-0.113	-0.363	-0.113	-0.363
**H15**	0.379	0.383	0.382	0.382	0.304	0.296	0.304	0.296
**C16**	-0.415	-0.352	-0.407	-0.356	-0.367	0.025	-0.367	0.026
**H17**	0.387	0.386	0.386	0.385	0.286	0.276	0.286	0.276
**H18**	0.387	0.386	0.386	0.385	0.387	0.277	0.287	0.277
**H19**	0.386	0.379	0.386	0.379	0.266	0.268	0.266	0.268
**C20**	-0.029	-0.060	-0.029	-0.052	1.196	1.201	1.196	1.200
**C21**	-0.418	-0.434	-0.418	-0.427	-1.630	-1.864	-1.630	-1.862

**C22**	-0.407	-0.423	-0.407	-0.416	-0.497	-0.464	-0.496	-0.466
**C23**	0.386	-0.391	-0.386	-0.389	0.338	0.379	0.338	0.379
**H24**	0.371	0.372	0.370	0.371	0.226	0.196	0.226	0.196
**C25**	-0.385	-0.392	-0.385	-0.389	0.129	0.186	0.129	0.185
**H26**	0.374	0.371	0.374	0.373	0.280	0.284	0.280	0.284
**C27**	-0.424	-0.449	-0.424	-0.440	-0.881	-0.891	-0.881	-0.891
**H28**	0.387	0.387	0.386	0.387	0.298	0.300	0.298	0.284
**H29**	0.386	0.386	0.386	0.386	0.291	0.291	0.291	0.291
**H30**	0.386	0.384	0.386	0.385	0.251	0.250	0.251	0.250

Table S2. Estimation of polarity of **APT** in Vacuum and in solvents of varying polarities

**APT**	VACUUM		WATER		METHANOL		ACETONITRILE	
Atom	Ground state	Excited state	Ground state	Excited state	Ground state	Excited state	Ground state	Excited state
**C1**	-0.204	-0.094	-0.246	-0.733	-0.244	-0.738	-0.244	-0.737
**C2**	0.185	-0.588	0.128	0.966	0.129	0.970	0.128	0.969
**C3**	-0.449	-0.005	-0.314	-0.483	-0.318	-0.484	-0.317	-0.484
**C4**	-0.075	-0.155	-0.111	0.022	-0.110	0.022	-0.110	0.022
**C5**	-0.053	-0.287	-0.066	-0.165	-0.066	-0.170	-0.066	-0.169
**C6**	-0.100	-0.291	-0.150	-0.037	-0.149	-0.035	-0.149	-0.036
**N7**	-0.281	-0.258	-0.264	0.158	-0.264	0.159	-0.264	0.158
**C8**	0.242	0.264	0.165	-1.366	0.167	-1.366	0.167	-1.366
**N9**	-0.264	-0.104	-0.209	-0.160	-0.211	-0.160	-0.211	-0.160
**C10**	-0.307	-0.193	-0.315	0.588	0.318	0.579	-0.318	0.581
**N11**	-0.125	-0.186	-0.189	-0.200	-0.187	-0.196	-0.187	-0.196
**N12**	0.008	0.160	-0.145	-0.154	-0.140	-0.149	-0.141	-0.150

**C13**	0.699	1.132	0.640	0.675	0.643	0.681	0.642	0.681
**C14**	-0.207	-0.181	-0.214	-0.175	-0.213	-0.173	-0.214	-0.173
**C15**	0.215	0.275	0.189	0.273	0.190	0.266	0.190	0.267
**C16**	-0.254	-0.372	-0.243	-0.312	-0.242	-0.314	-0.242	-0.313
**C17**	-0.016	0.300	-0.158	0.153	-0.154	0.157	-0.154	0.156
**C18**	-0.963	-1.700	-0.661	-1.168	-0.671	-1.164	-0.669	-1.165
**H19**	0.153	0.090	0.161	0.213	0.161	0.214	0.161	0.214
**H20**	0.109	0.100	0.136	0.163	0.135	0.163	0.135	0.163
**H21**	0.124	0.118	0.143	0.166	0.142	0.166	0.142	0.166
**H22**	0.117	0.095	0.136	0.162	0.136	0.162	0.136	0.162
**H23**	0.115	0.087	0.130	0.157	0.130	0.156	0.130	0.156
**H24**	0.351	0.397	0.366	0.383	0.366	0.382	0.366	0.382
**H25**	0.361	0.416	0.135	0.126	0.134	0.126	0.134	0.126
**H26**	0.116	0.137	0.161	0.154	0.162	0.154	0.162	0.154
**H27**	0.171	0.198	0.123	0.112	0.122	0.111	0.122	0.111
**H28**	0.101	0.116	0.146	0.154	0.146	0.153	0.146	0.153
**H29**	0.127	0.167	0.139	-0.023	0.138	-0.023	0.138	-0.023
**H30**	0.104	-0.040	0.386	0.351	0.386	0.350	0.386	0.351

Table S3. Estimation of polarity of **NpTP** in Vacuum and in solvents of varying polarities

**NpTP**	VACUUM		WATER		METHANOL		ACETONITRILE	
Atom	Ground state	Excited state	Ground state	Excited state	Ground state	Excited state	Ground state	Excited state
C1	-0.398	-0.399	-0.351	-0.307	-0.408	-0.435	-0.351	-0.435
C2	-0.373	-0.376	-0.270	-0.381	-0.307	-0.250	-0.270	-0.250
C3	-0.387	-0.398	-0.343	-0.402	-0.304	-0.305	-0.343	-0.305

C4	-0.034	-0.044	-0.076	-0.040	0.480	0.440	0.076	0.440
C5	-0.379	-0.371	-0.287	-0.369	-0.390	-0.288	-0.287	-0.288
C6	-0.019	-0.038	-0.180	-0.037	-1.008	-0.905	-0.180	-0.904
C7	-0.369	-0.353	-0.327	-0.351	-0.104	-0.072	-0.327	-0.072
C8	-0.386	-0.401	-0.269	-0.405	-0.551	-0.505	-0.269	-0.505
C9	-0.410	-0.397	-0.190	-0.399	-0.729	-0.728	-0.189	-0.728
C10	-0.044	-0.050	-0.014	-0.047	1.179	0.881	-0.015	0.881
C11	0.132	0.112	-0.272	0.102	-0.821	-0.940	-0.272	-0.940
N12	-0.463	-0.444	-0.438	-0.430	-0.354	-0.330	-0.438	-0.330
C13	-0.046	0.002	0.112	0.002	-0.250	-0.128	0.112	-0.128
N14	0.056	0.081	0.139	0.076	0.379	0.390	0.139	0.390
N15	-0.302	-0.339	-0.409	-0.337	-0.451	0.485	-0.409	0.485
C16	0.408	0.423	0.430	0.423	-0.149	-0.204	0.430	-0.204
C17	0.516	0.509	0.442	0.519	-0.375	-0.318	0.442	-0.318
C18	-0.454	-0.447	-0.434	-0.453	0.368	0.384	-0.434	0.385
C19	-0.510	-0.512	-0.433	-0.516	-0.109	-0.147	-0.433	-0.147
C20	-0.477	0.481	-0.411	-0.480	-0.644	-0.671	-0.411	-0.671
C21	-0.431	-0.428	-0.337	-0.429	-0.317	-0.282	-0.337	-0.282
O22	-0.744	-0.744	-0.785	-0.745	-0.219	-0.219	-0.785	-0.219
H23	0.386	0.384	0.333	0.384	0.337	0.336	0.333	0.336
H24	0.382	0.381	0.344	0.380	0.296	0.295	0.344	0.295
H25	0.386	0.387	0.334	0.387	0.332	0.331	0.334	0.331
H26	0.382	0.379	0.342	0.379	0.338	0.340	0.342	0.340
H27	0.382	0.380	0.341	0.379	0.318	0.317	0.341	0.317

H28	0.371	0.373	0.332	0.373	0.341	0.340	0.332	0.340
H29	0.374	0.369	0.371	0.369	0.070	0.035	0.371	0.035
H30	0.385	0.383	0.377	0.383	0.424	0.440	0.377	0.440
H31	0.387	0.385	0.352	0.386	0.253	0.251	0.352	0.251
H32	0.390	0.389	0.345	0.390	0.322	0.322	0.345	0.322
H33	0.388	0.388	0.337	0.388	0.349	0.350	0.337	0.350
H34	0.388	0.386	0.338	0.387	0.335	0.334	0.338	0.334
H35	0.512	0.511	0.556	0.512	0.456	0.456	0.556	0.456

Table S4. Estimation of polarity of **PhTP** in Vacuum and in solvents of varying polarities

**PhTP**	VACUUM		WATER		METHANOL		ACETONITRILE	
Atom	Ground state	Excited state	Ground state	Excited state	Ground state	Excited state	Ground state	Excited state
C1	-0.393	-0.394	-0.393	-0.391	-0.755	-0.762	-0.755	-0.762
C2	-0.385	-0.397	-0.384	-0.404	-1.144	-1.119	-1.144	-1.119
C3	-0.389	-0.399	-0.389	-0.404	-0.290	-0.293	-0.290	-0.293
C4	-0.008	-0.004	-0.008	0.001	0.851	0.954	0.851	0.954
C5	-0.379	-0.376	-0.378	-0.373	-0.260	-0.248	-0.260	-0.248
C6	-0.031	-0.041	-0.030	-0.043	0.199	0.071	0.199	0.071
C7	0.003	0.002	0.003	0.008	0.837	0.860	0.837	0.860
C8	0.001	-0.016	0.001	-0.018	0.808	0.840	0.808	0.840
C9	-0.430	-0.427	-0.430	-0.429	-0.563	-0.621	-0.563	-0.620
C10	0.008	0.010	0.009	0.013	0.932	0.786	0.932	0.786
C11	-0.386	-0.387	-0.385	-0.391	-1.052	-0.932	-1.052	-0.932
C12	-0.386	-0.382	-0.386	-0.380	-0.444	-0.451	-0.444	-0.451

C13	-0.388	-0.394	-0.387	-0.395	-0.528	-0.597	-0.528	-0.597
C14	-0.422	-0.409	-0.421	-0.406	-1.130	-1.106	-1.130	-1.106
C15	0.154	0.135	0.155	0.118	-0.807	-0.873	-0.807	-0.873
N16	-0.468	-0.454	-0.473	-0.436	-0.735	-0.714	-0.735	-0.713
C17	-0.046	-0.001	-0.047	0.001	-0.561	-0.446	-0.561	-0.446
N18	0.059	0.079	0.054	0.069	0.926	0.913	0.926	0.912
N19	-0.305	-0.333	-0.300	-0.322	0.414	0.448	0.414	0.448
C20	0.409	0.424	0.408	0.417	-0.028	-0.044	-0.028	-0.043
C21	0.516	0.507	0.514	0.517	-0.447	-0.421	-0.447	-0.421
C22	-0.454	-0.446	-0.455	-0.453	0.425	0.407	0.425	0.407
C23	-0.509	-0.511	-0.509	-0.513	-0.168	-0.170	-0.168	-0.170
C24	-0.477	-0.481	-0.476	-0.478	-0.636	-0.621	-0.636	-0.621
C25	-0.430	-0.426	-0.428	-0.428	-0.284	-0.282	-0.284	-0.282
O26	-0.744	-0.744	-0.744	-0.744	-0.210	-0.210	-0.210	-0.210
H27	0.386	0.385	0.386	0.383	0.348	0.346	0.348	0.346
H28	0.382	0.382	0.382	0.382	0.255	0.255	0.255	0.255
H29	0.387	0.387	0.387	0.387	0.332	0.332	0.332	0.332
H30	0.383	0.381	0.382	0.380	0.350	0.351	0.350	0.351
H31	0.375	0.371	0.375	0.371	-0.054	-0.081	-0.054	-0.081
H32	0.383	0.382	0.383	0.381	0.227	0.228	0.227	0.228
H33	0.387	0.386	0.387	0.386	0.352	0.351	0.352	0.351
H34	0.387	0.387	0.386	0.387	0.340	0.340	0.340	0.340
H35	0.359	0.358	0.359	0.357	0.308	0.300	0.308	0.300
H36	0.386	0.384	0.386	0.384	0.499	0.515	0.499	0.515

H37	0.388	0.386	0.388	0.388	0.232	0.231	0.232	0.231
H38	0.390	0.389	0.390	0.390	0.323	0.323	0.323	0.323
H39	0.388	0.388	0.388	0.388	0.353	0.354	0.353	0.354
H40	0.388	0.386	0.388	0.387	0.332	0.332	0.332	0.332
H41	0.512	0.511	0.513	0.512	0.454	0.455	0.454	0.455

Table S5. Estimation of polarity of **PTP** in Vacuum and in solvents of varying polarities

**PTP**	VACUUM		WATER		METHANOL		ACETONITRILE	
Atom	Ground state	Excited state	Ground state	Excited state	Ground state	Excited state	Ground state	Excited state
C1	-0.412	-0.413	-0.411	-0.412	-0.704	-0.668	-0.704	-0.668
C2	-0.385	-0.389	-0.385	-0.389	-0.106	-0.195	-0.106	-0.195
C3	-0.384	-0.385	-0.383	-0.386	-0.147	-0.227	-0.147	-0.227
C4	-0.402	-0.404	-0.402	-0.404	0.813	0.867	0.813	0.867
C5	-0.404	-0.401	-0.404	-0.402	-3.019	-2.923	-3.019	-2.923
C6	-0.050	-0.054	-0.048	-0.051	1.789	1.636	1.789	1.636
C7	0.143	0.145	0.144	0.140	-1.448	-1.458	-1.448	-1.458
N8	-0.450	-0.428	-0.451	-0.426	-0.304	-0.326	-0.304	-0.326
C9	0.043	-0.036	-0.043	-0.037	0.463	0.673	0.463	0.673
N10	0.097	0.140	0.097	0.144	0.421	0.481	0.421	0.481
N11	-0.281	-0.311	-0.281	-0.328	0.365	0.355	0.365	0.355
C12	0.396	0.396	0.395	0.413	-0.146	-0.288	-0.146	-0.288
C13	0.502	0.555	0.496	0.549	-0.199	-0.123	-0.199	-0.123
C14	-0.450	-0.455	-0.449	-0.460	0.448	0.417	0.448	0.417
C15	-0.509	-0.540	-0.508	-0.540	0.113	0.061	0.113	0.061

C16	-0.477	-0.485	-0.475	-0.487	-0.872	-0.880	-0.872	-0.880
C17	-0.425	-0.437	-0.424	-0.432	-0.193	-0.209	-0.193	-0.209
O18	-0.777	-0.790	-0.777	-0.776	-0.521	-0.506	-0.521	-0.506
H19	0.382	0.377	0.382	0.377	0.397	0.384	0.397	0.384
H20	0.471	0.473	0.470	0.472	0.541	0.556	0.541	0.556
H21	0.379	0.378	0.379	0.378	0.123	0.120	0.123	0.120
H22	0.387	0.387	0.386	0.386	0.316	0.386	0.316	0.316
H23	0.386	0.385	0.386	0.385	0.256	0.385	0.256	0.256
H24	0.386	0.386	0.386	0.386	0.285	0.386	0.286	0.285
H25	0.372	0.372	0.372	0.371	0.280	0.371	0.279	0.280
H26	0.387	0.383	0.387	0.384	0.212	0.384	0.199	0.212
H27	0387	0.383	0387	0.384	0.279	0.384	0.284	0.279
H28	0.386	0.385	0.386	0.384	0.275	0.384	0.276	0.275
H29	0.388	0.386	0.388	0.387	0.289	0.387	0.290	0.289

Deconvoluted fluorescence emission spectrum of PhTP in ACN

Spectral deconvolution method: The fluorescence spectra were deconvoluted into overlapping Gaussian components using the fitting algorithm in the FluorEssence software (integrated with the Fluoromax Plus). Adjustable parameters such as the band center and width were optimized to obtain the minimum number of reproducible components. Multiple fits were performed with varying initial parameters to assess the range of statistically equivalent solutions. Among the different spectral resolutions, a "good fit" was identified based on several criteria, including a minimal goodness-of-fit value (χ^2) and close superposition of the reconstructed curves with the experimentally measured spectra.[1]

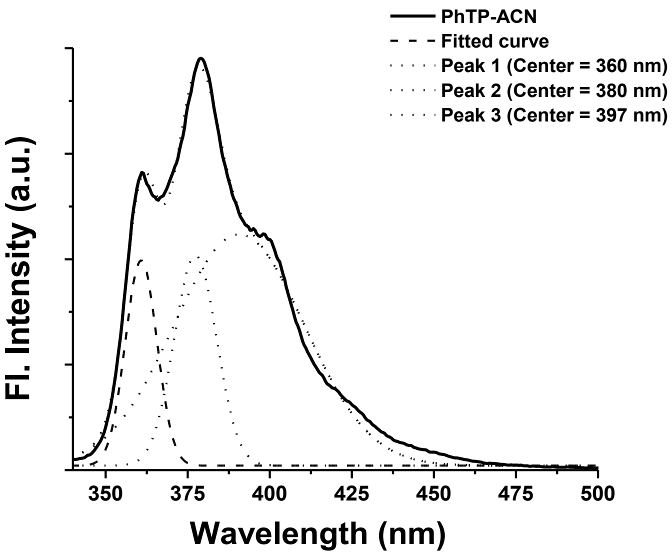

Parameters

	Value	Error
y0	4.2715048681003	0.35694323027532
xc1	360.79253179487	0.091586357297284
w1	9.6115085064419	0.19639142655147
A1	2348.031128813	74.834724361456
xc2	377.33142414528	0.10199789921298
w2	12.890525043776	0.26478651110159
A2	3223.3923763334	107.00139833939
xc3	390.67383476724	0.35404486703437
w3	39.810979887049	0.41774595762933
A3	10937.924381429	182.09678255839

Peaks

	Area	Center	Width	Height
1	2348.031128813	360.79253179487	9.6115085064419	194.91818424848
2	3223.3923763334	377.33142414528	12.890525043776	199.51825094416
3	10937.924381429	390.67383476724	39.810979887049	219.21593027682

Statistics

	Statistics
DF	556
COD (R^2)	0.99615580850264
ReducedChiSq	42.248450706658

Figure S3. Resolved fluorescence emission spectra of PhTP (2×10^{-5} M) in ACN. Solid line denotes the experimental spectra, the dash-dot line represents the resolved spectra, and the dotted lines represent the convoluted spectra from the resolved bands.

Table S6. Absorption and emission maxima, Stokes shifts, and the quantum yield (Φ) of the 1,2,3-triazoles in various solvents.

The Stokes shift (cm^{-1}) is defined as $\Delta\bar{\nu} = \bar{\nu}_{abs.max} - \bar{\nu}_{em.max}$ where $\bar{\nu}_{abs.max}$, and $\bar{\nu}_{em.max}$ are the wavenumbers (cm^{-1}) of the absorption and emission maxima, respectively.

Quantum yield calculation of the unknown sample (S):

$$\Phi_S = \Phi_{ref} \cdot \frac{(Area\ under\ fluorescence)_S}{(Area\ under\ fluorescence)_{ref}} \cdot \frac{(Absorbance)_{ref}}{(Absorbance)_S} \cdot \frac{\eta_S^2}{\eta_{ref}^2} \qquad \text{(Equation S1)}$$

To correlate the excitation maxima of the individual triazoles (270-330 nm), quinine sulfate was excited at 270 nm (Φ_{ref} at 270 nm = 0.43 ± 0.03) and 310 (Φ_{ref} at 310 nm = 0.50 ± 0.04) nm to assess the quantum yield at these maximum excitation wavelengths, which was used as the reference for the triazoles.

S6a. PTP:

Solvent	λ_{max}^{em} (emission scans) (nm)	λ_{max}^{abs} (absorption spectra) (nm)	Stokes shift (cm^{-1})	Quantum yield
Hexane	-	303.3	-	0.0049 ± 0.0001
Acetonitrile	-	291.5	-	0.0017 ± 0.0001
Methanol	403.0	284.4	10348	0.033 ± 0.002
Ethanol	437.0	289.9	11611	0.14 ± 0.04
Water	380.0	281.7	91833	0.028 ± 0.003

S6b. PhTP:

Solvent	λ_{max}^{em} (emission scans) (nm)	λ_{max}^{abs} (absorption spectra) (nm)	Stokes shift (cm^{-1})	Quantum yield
Hexane	379.6	304.0	6551	0.090 ± 0.008
Acetonitrile	377.7	300.0	6857	0.12 ± 0.01
Methanol	377	298.04	7027	0.17 ± 0.02
Ethanol	377.6	298.6	7007	0.22 ± 0.04
Water	376.2	292.0	7665	0.019 ± 0.002

S6c. ADT:

Solvent	$\lambda_{\max}^{em}$ (emission scans) (nm)	$\lambda_{\max}^{abs}$ (absorption spectra) (nm)	Stokes shift (cm^{-1})	Quantum yield
Acetonitrile	372.7	268.9	10357	0.00075 ± 0.00007
Methanol	357.5	267.3	9439	0.00016 ± 0.00002
Water	401.6	260.1	13546	0.00061 ± 0.00001

S6d. APT:

Solvent	$\lambda_{\max}^{em}$ (emission scans) (nm)	$\lambda_{\max}^{abs}$ (absorption spectra) (nm)	Stokes shift (cm^{-1})	Quantum yield
Heptane	408	268	12804	0.04 ± 0.003
Acetonitrile	390	268	11672	0.61 ± 0.04
Methanol	420	265	13926	0.09 ± 0.002
Ethanol	411	268	12983	0.03 ± 0.004
Water	435	264	14890	0.009 ± 0.0002

Concentration-dependent controls of APT in ACN

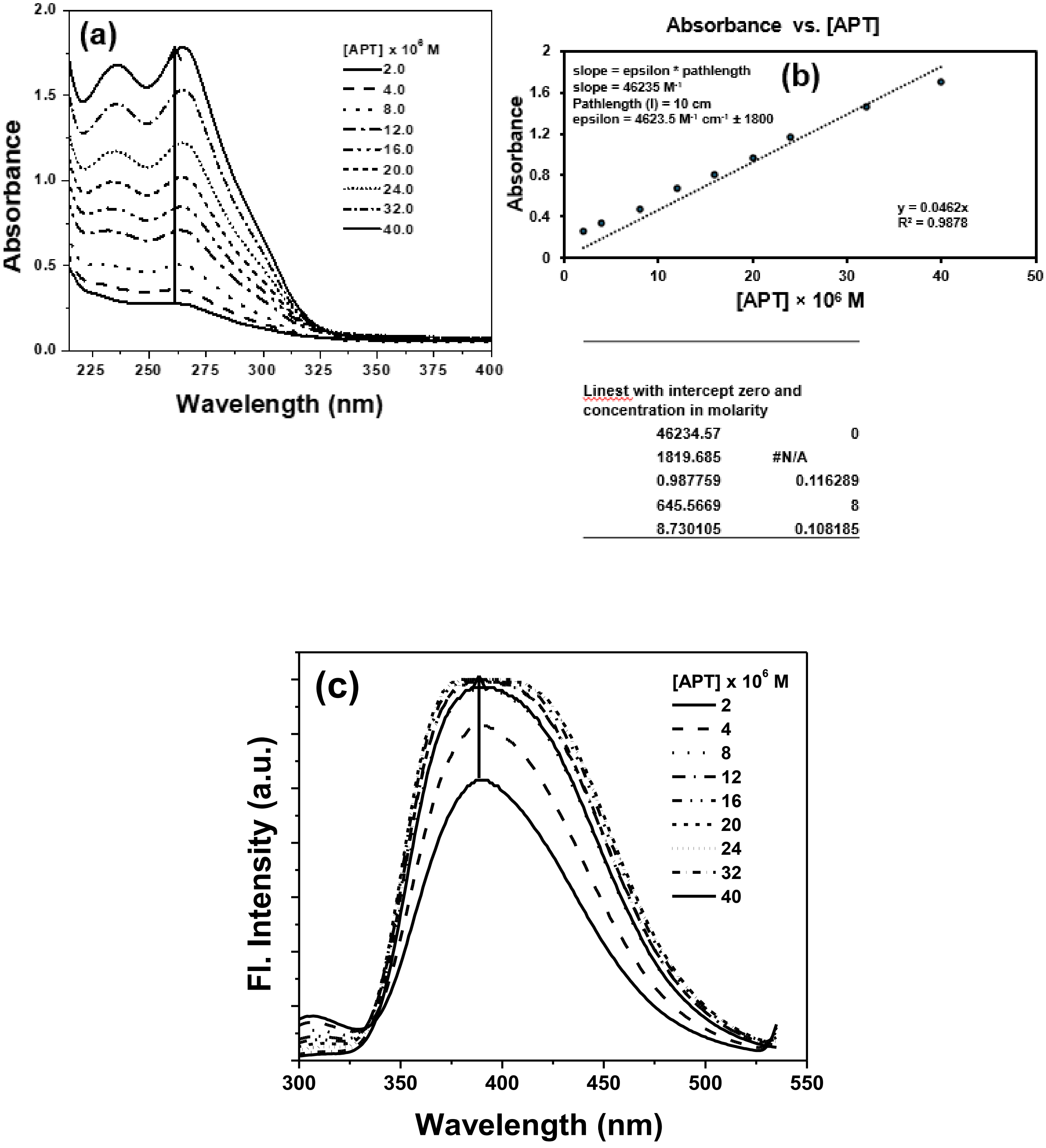

Figure S4. Concentration-dependent controls for absorption vs concentration of APT, (a) absorption spectra, (b) absorption linearity, and (c) emission scans for the same concentration range of APT in ACN (λ_{exc} = 270 nm).

pH variation studies of PTP, PhTP, and APT

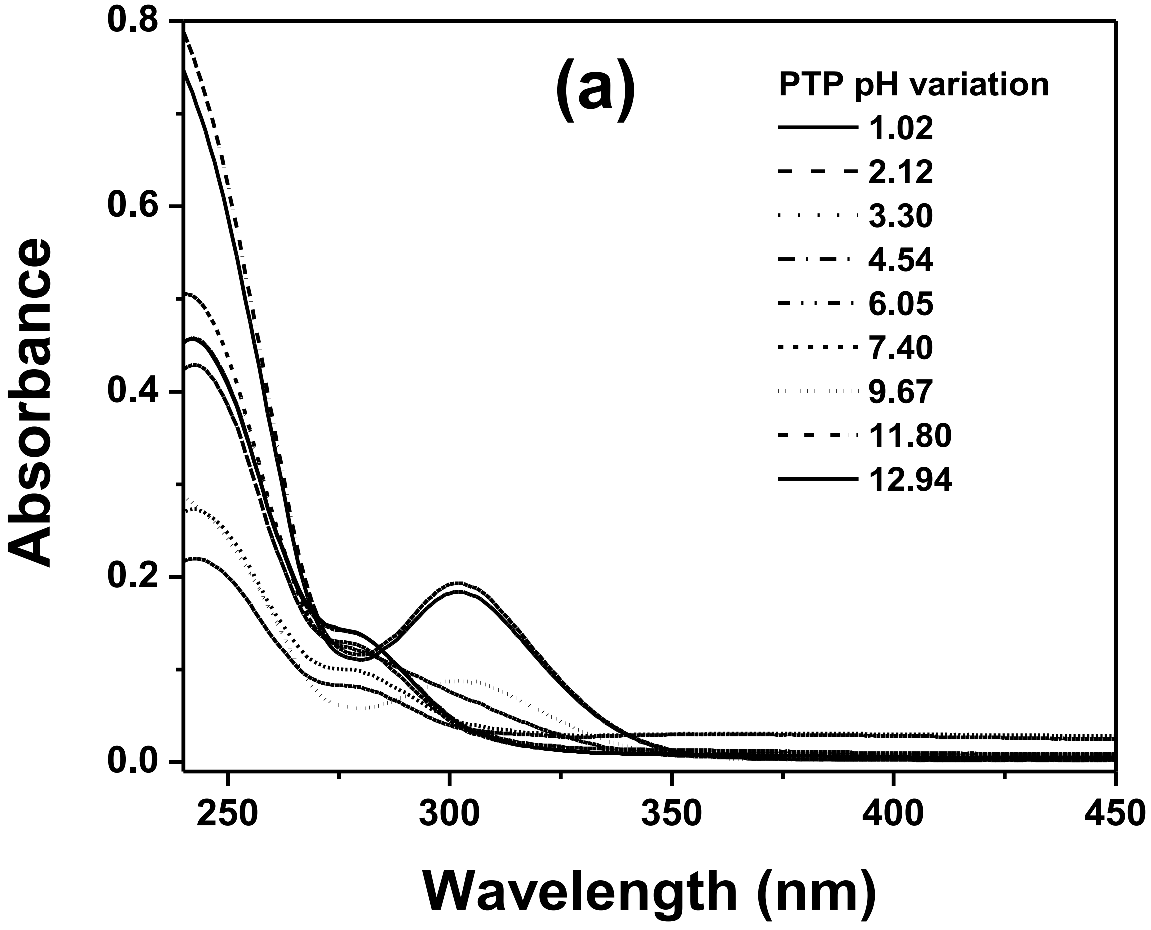

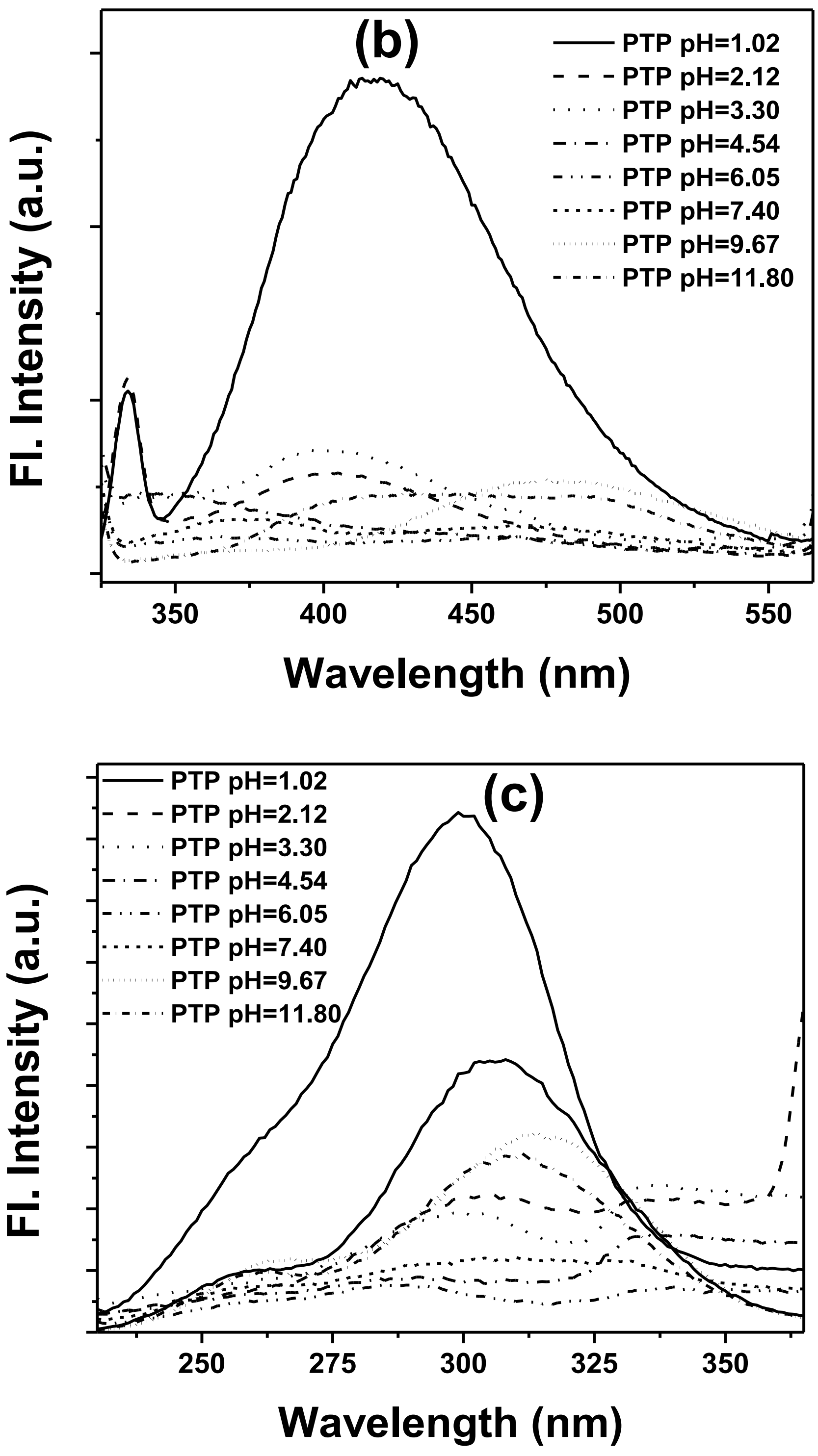

Figure S5. (a) UV-Vis absorption, (b) emission (λ_{exc} = 290 nm), and (c) excitation (λ_{em} = 450 nm) spectra of PTP (2 × 10^{-5} M) at different pH levels.

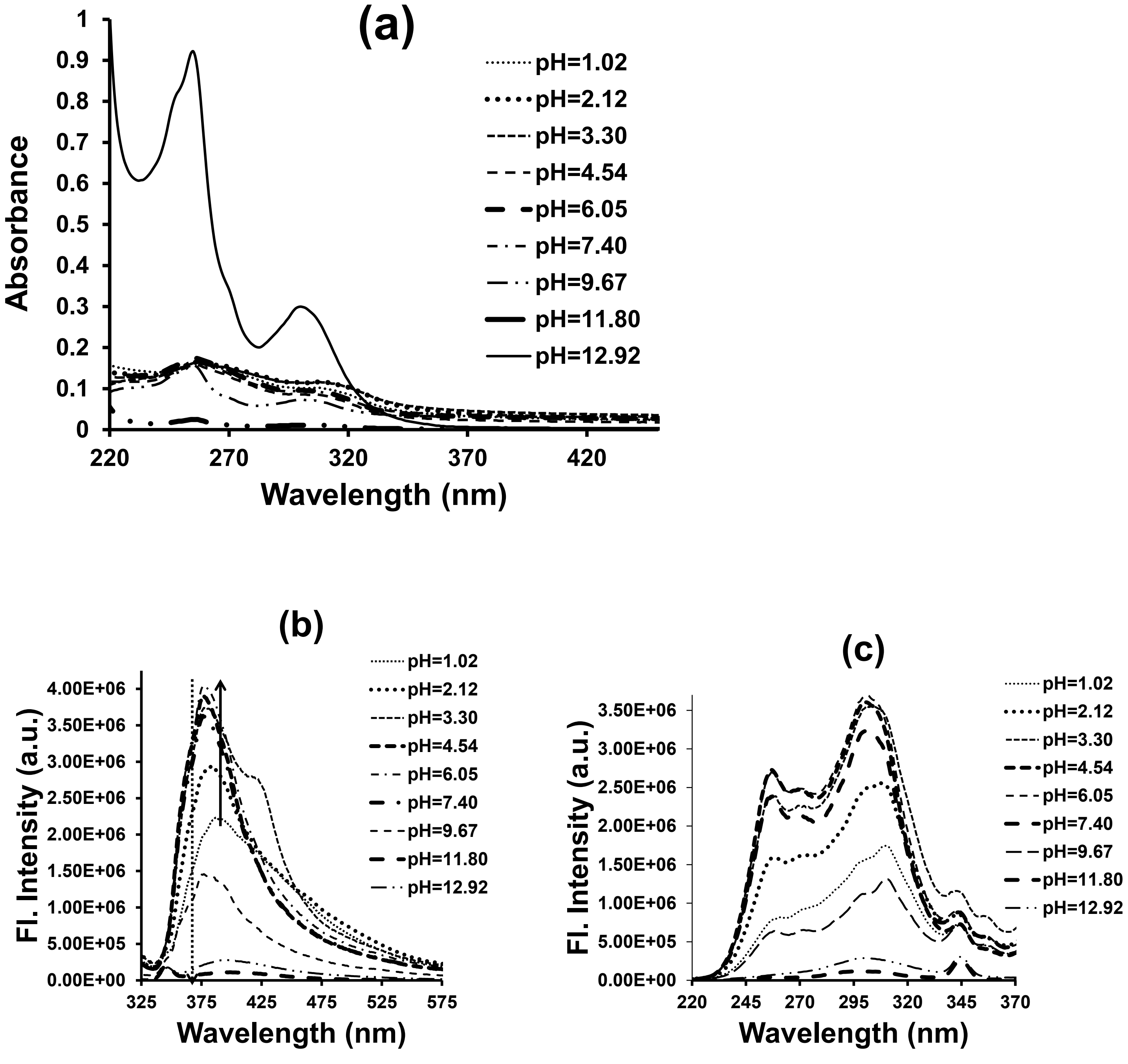

Figure S6. (a) UV-Vis absorption, (b) emission (λ_{exc} = 300 nm), and (c) excitation (λ_{em} = 390 nm) spectra of PhTP (2 × 10^{-5} M) at different pH levels

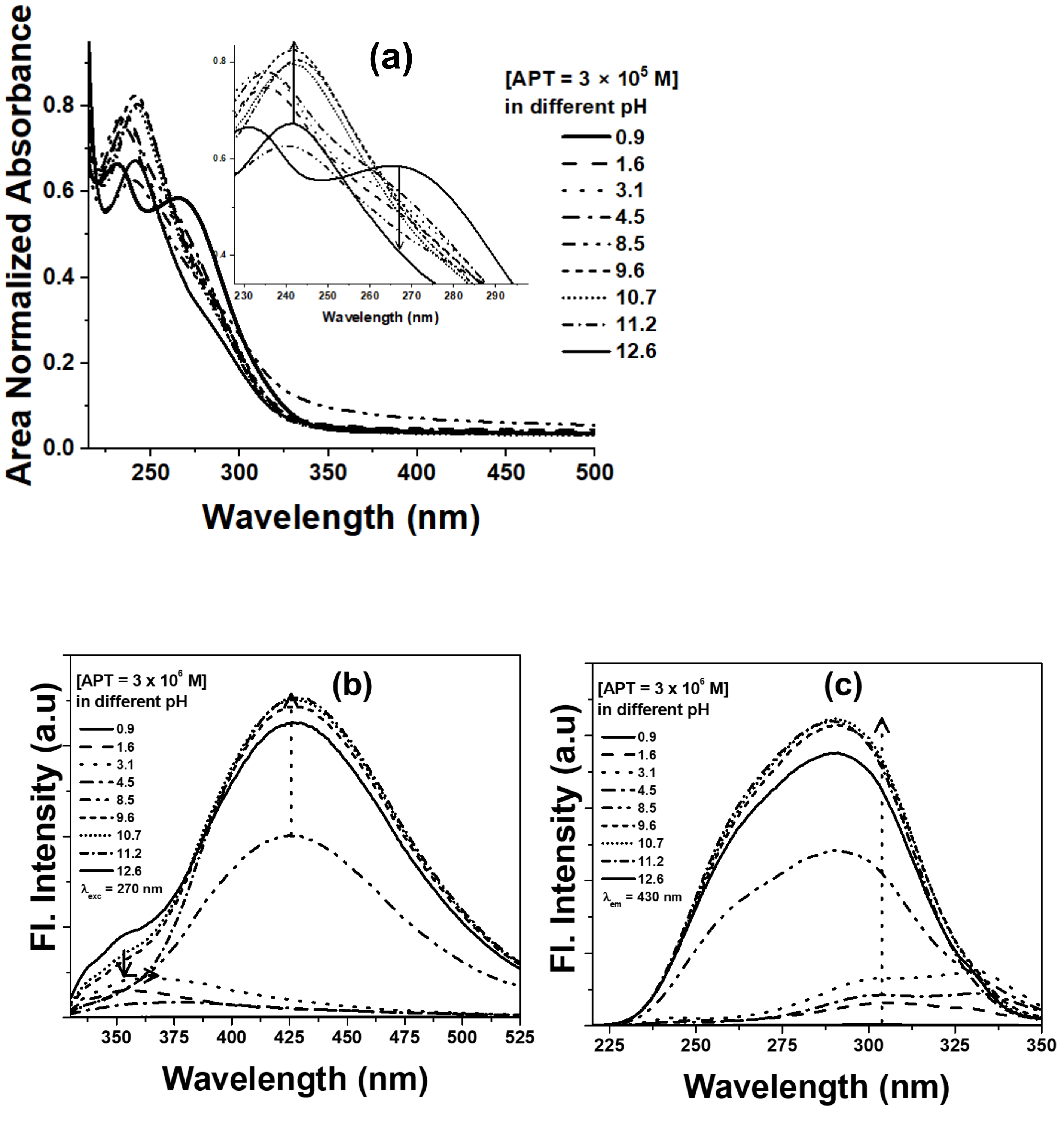

Figure S7. (a) UV-Vis absorption, (b) emission (λ_{exc} = 270 nm), and (c) excitation (λ_{em} = 430 nm) spectra of APT at different pH levels

Excitation and emission scans of 1,2,3-triazoles in ACN

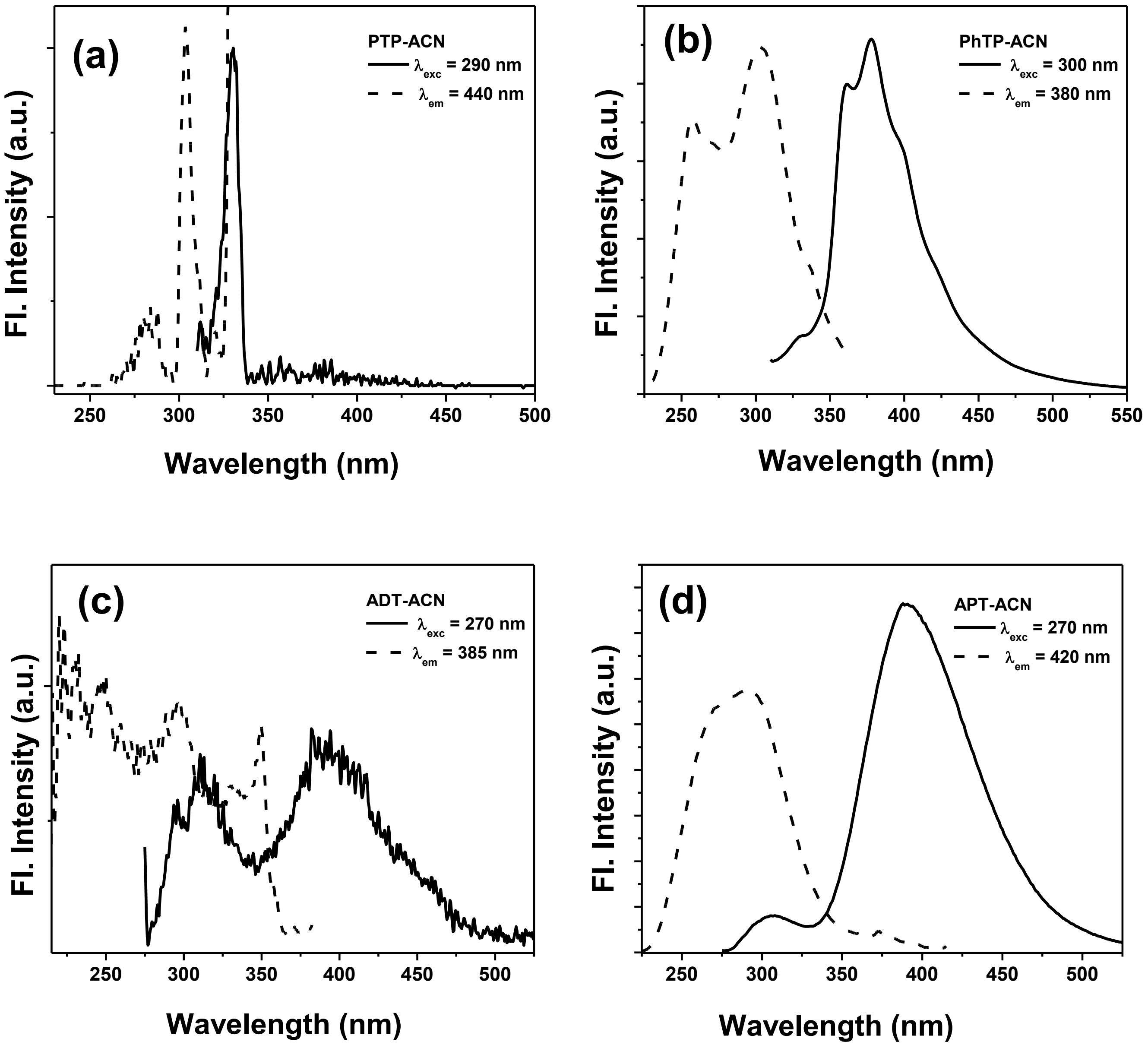

Figure S8. Emission (solid lines) and excitation (dashed lines) scans for (a) PTP (2×10^{-5} M), (b) PhTP (1×10^{-5} M), (c) ADT (2×10^{-6} M), and (d) APT (2×10^{-6} M) in ACN.

Excitation and emission scans of PhTP in hexane and ethanol

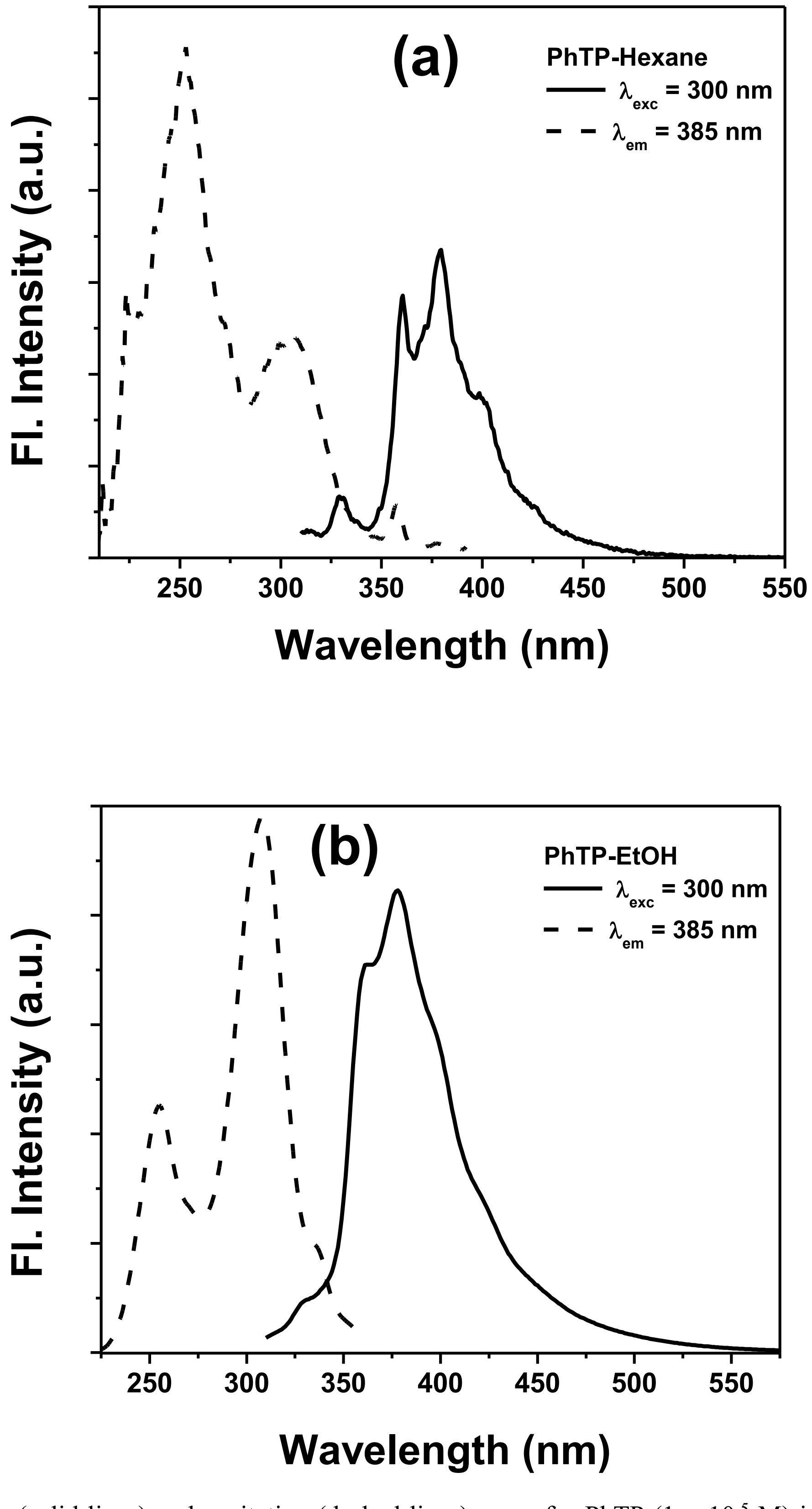

Figure S9. Emission (solid lines) and excitation (dashed lines) scans for PhTP (1×10^{-5} M) in (a) hexane and (b) ethanol.

Absorption, emission, and excitation scans of APT in glycerol-water mixture

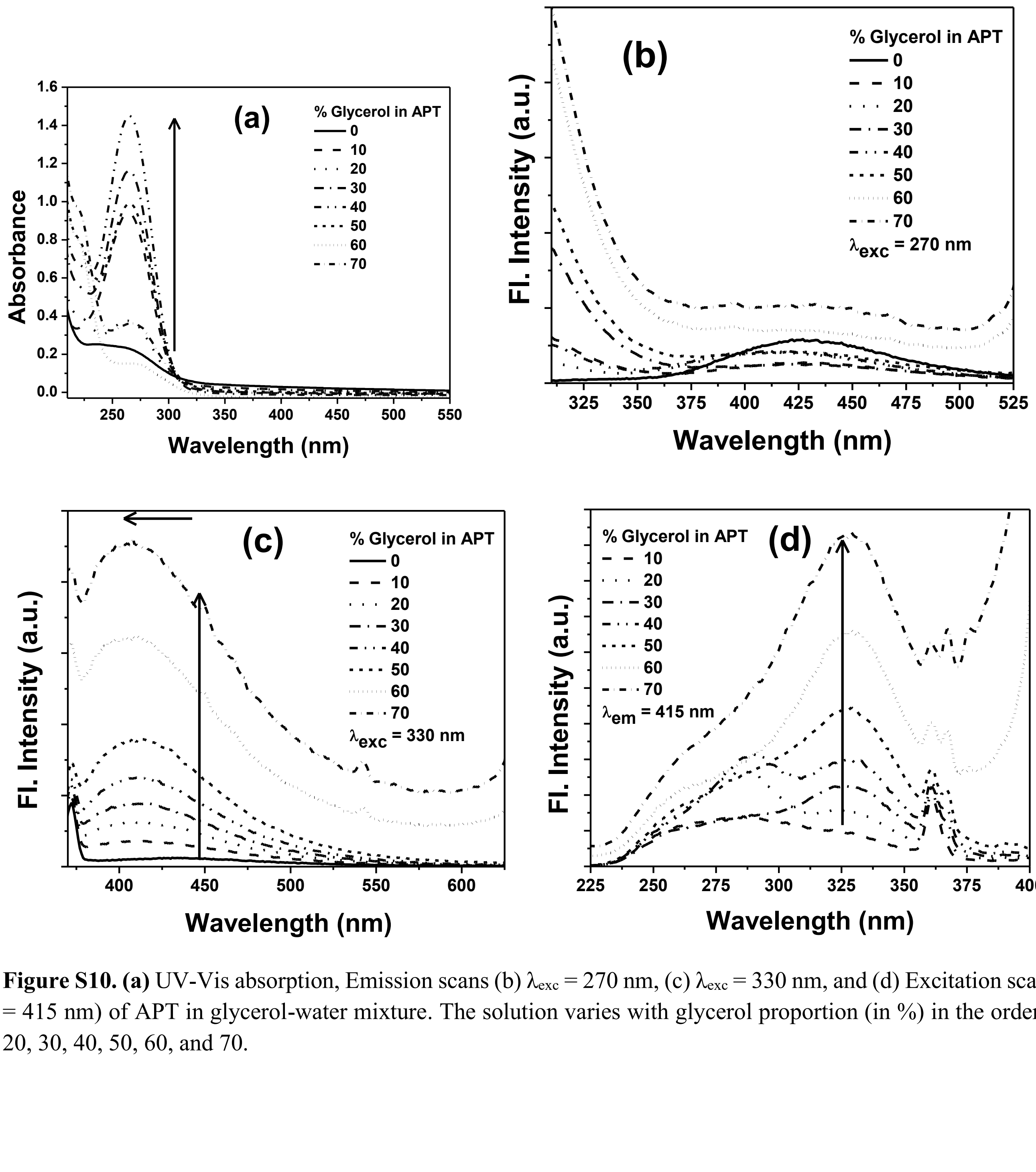

Figure S10. (a) UV-Vis absorption, Emission scans (b) λ_{exc} = 270 nm, (c) λ_{exc} = 330 nm, and (d) Excitation scans (λ_{em} = 415 nm) of APT in glycerol-water mixture. The solution varies with glycerol proportion (in %) in the order 0, 10, 20, 30, 40, 50, 60, and 70.

Emission intensity vs. time of PhTP

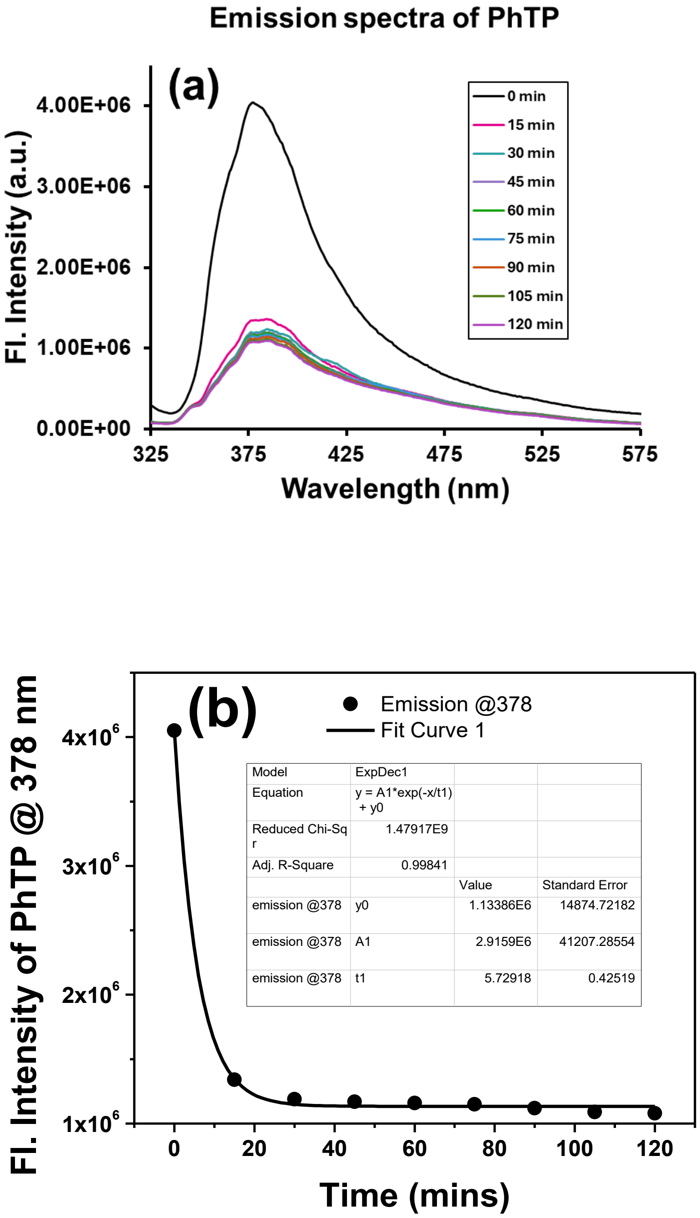

Estimation of decay constant from the fitted plot = 0.175 mins^{-1}

Estimation of half-life ($t_{1/2}$) from decay constant = 3.96 mins

Figure S11. (a) Emission scans of PhTP (λ_{exc} = 300 nm) in pH 6.05 at different times in air, and (b) Intensity vs. time plot.

References:

(1) Ghosh, D.; Sarkar, D.; Chattopadhyay, N. Intramolecular Charge Transfer Promoted Fluorescence Transfer: A Demonstration of Re-Absorption of the Donor Fluorescence by the Acceptor. *J. Mol. Liq.* **2010**, *156* (2), 131–136. https://doi.org/10.1016/j.molliq.2010.06.006.
